# Airborne particulate matter and diesel engine exhaust on infrastructure construction sites in the Copenhagen metropolitan area

**DOI:** 10.1093/annweh/wxae062

**Published:** 2024-09-17

**Authors:** Patrick L Ferree, Merve Polat, Jakob K Nøjgaard, Keld A Jensen

**Affiliations:** The National Research Centre for the Working Environment, Lersø Parkallé 105, Copenhagen DK-2100, Denmark; The National Research Centre for the Working Environment, Lersø Parkallé 105, Copenhagen DK-2100, Denmark; The National Research Centre for the Working Environment, Lersø Parkallé 105, Copenhagen DK-2100, Denmark; The National Research Centre for the Working Environment, Lersø Parkallé 105, Copenhagen DK-2100, Denmark

**Keywords:** black carbon, construction industry, elemental carbon, heavy-duty diesel engine exhaust, particulate matter, ultrafine particles

## Abstract

Diesel engine exhaust (DEE) is carcinogenic and potentially hazardous for those working in close proximity to diesel-powered machines. This study characterizes workplace exposure to DEE and its associated particulate matter (PM) during outdoor construction activities. We sampled at 4 construction sites in the Copenhagen metropolitan area. We used portable constant-flow pumps and quartz-fiber filters to quantify personal exposure to elemental carbon (EC), and used real-time instruments to collect activity-based information about particle number and size distribution, as well as black carbon (BC) concentration. Full-shift measurements of EC concentration ranged from < 0.3 to 6.4 µg/m^3^. Geometric mean (GM) EC exposure was highest for ground workers (3.4 µg/m^3^ EC; geometric standard deviation, GSD = 1.3), followed by drilling rig operators (2.6 µg/m^3^ EC; GSD = 1.4). Exposure for non-drilling-rig machine operators (1.2 µg/m^3^ EC; GSD = 2.9) did not differ significantly from background (0.9 µg/m^3^ EC; GSD = 1.7). The maximum 15-min moving average concentration of BC was 17 µg/m^3^, and the highest recorded peak concentration was 44 µg/m^3^. In numbers, the particle size distributions were dominated by ultrafine particles ascribed to DEE and occasional welding activities at the sites. The average total particle number concentrations (PNCs) measured in near-field and far-field positions across all worksites were 10,600 (GSD = 3.0) and 6,000 (GSD = 2.8)/cm^3^, respectively. Sites with active drilling rigs saw significantly higher average total PNCs at their near-field stations (13,600, 32,000, and 9,700/cm^3^; GSD = 2.4, 3.4, and 2.4) than sites without (4,700/cm^3^; GSD = 1.6). Overall, the DEE exposures at these outdoor construction sites were below current occupational exposure limits for EC (10 µg/m^3^ in Denmark; 50 µg/m^3^ in the European Union), but extended durations of exposure to the observed DEE levels may still be a health risk.

**What’s Important About This Paper?**

There is currently a great deal of interest in lowering occupational exposure limits (OELs) to diesel engine exhaust (DEE), but there are few recent studies of DEE exposure on outdoor construction sites. This study evaluated personal exposure to DEE during typical workdays for machine operators and ground workers, as well as time-course information regarding the concentration of fine and ultrafine particles emitted from construction machinery. Though DEE exposures were generally below Danish and European OELs, our results will be useful for assessing the feasibility and impact of further reduction in the OELs.

## Introduction

Diesel engines remain widespread, and their emissions (DEE) contribute significantly to air pollution in urban and workplace environments. This is especially true for industries like construction, transportation, railways, shipping, and mining, where the nature of the work often mandates the deployment of heavy-duty machines (Pronk et al. 2009). Earlier studies paved the way for DEE regulation, but 2 occupational epidemiological studies in particular were critical for the evaluation and eventual IARC classification of DEE as carcinogenic (Garshick et al. 2012; Silverman et al. 2012; IARC 2014; HEI Diesel Epidemiology Panel 2015). Both studies linked long-term DEE exposure with an increased risk of developing lung cancer, but DEE has also been linked to bladder cancer (Koutros et al. 2020) and cardiovascular disease (Brook et al. 2010). Given the ubiquity of diesel engines, these discoveries warrant DEE characterization on typical jobsites and their surroundings, as well as continual reassessment of appropriate occupational exposure limits (OELs).

DEE is a mixture of gases and particles that result from incomplete combustion of diesel fuel (Ris 2007), and its specific composition varies depending on the fuel, engine specifications, and workload. The gaseous exhaust generally includes water vapor; carbon, nitrogen, and sulfur oxides; as well as low-molecular-weight organic molecules, such as polycyclic aromatic hydrocarbons (McDonald et al. 2004). The particulate matter, on the other hand, is composed of elemental carbon (EC) clusters in the form of aggregated and agglomerated spheroidal graphite and other structures of unreacted carbon, as well as different trace metal compounds (Utsunomiya et al. 2004; Ris 2007; Liati et al. 2013; 2013; Karjalainen et al. 2016). DEE particles are generally in the fine and ultrafine size range (≤300 and 100 nm, respectively), with 80% to 90% by mass <2.5 µm and 50% to 90% by number <100 nm, and hence have the ability to penetrate deep into human lungs during respiration and deposit in the alveolar region (Ris 2007). Compared to their gasoline counterparts, diesel engines generally emit more PM relative to fuel mass and more EC content (Maricq 2007).

The heterogeneous nature of DEE complicates its quantification and, therefore, its regulation, but the solution has generally been to focus on full-shift measurements of EC concentration (Coble et al. 2010; Vermeulen et al. 2010). Currently, the European Union OEL for DEE is defined as 50 µg/m^3^ EC (Directive 2019), and in some countries, including Denmark and the Netherlands, it is as low as 10 µg/m^3^ (Arbejdstilsynet 2024). Some argue though that the limits should be lower (Vermeulen and Portengen 2016). The 1:1000 risk of developing lung cancer from work–life EC exposure has been set at 0.45 µg/m^3^ (Saber et al. 2018), and common cancer biomarkers are associated with exposure below current OELs (Wong et al. 2023). Hence, the Dutch Expert Committee on Occupational Safety (DECOS) recommends a respirable EC limit of 1.03 µg/m^3^ (Taxell and Santonen 2016), and the Finnish Institute of Occupational Health (FIOH) recommends 5 µg/m^3^ (Keefe et al. 2020).

Construction companies commonly use diesel-powered machines (Ringen et al. 2015), and thousands of construction workers worldwide are occupationally exposed to DEE. In Denmark, it is estimated that between 15,000 and 40,000 outdoor construction workers are exposed annually to DEE above background levels (Lassen et al. 2020). Most exposure studies on construction sites focus on particulate matter, such as silica dust (van Deurssen et al. 2014; Cothern et al. 2023; Gorman Ng et al. 2023). Fewer studies focus on DEE exposure, and most rely exclusively on filter-based sampling (Ziembicki et al. 2022), which overlooks acute exposures and the potential significance of ultrafine particles (Costa et al. 2014; Stone et al. 2017). An effort is underway to document workplace concentrations of ultrafine particles (Viitanen et al. 2017), and more studies of DEE exposure include complementary real-time particle measurements (Hedmer et al. 2017; da Silveira Fleck et al. 2020). In this paper, we investigate workplace exposures to EC, alongside temporal variation in black carbon (BC), particle number, and size distributions, in order to improve our knowledge of DEE exposures on larger construction sites in Denmark.

## Methods

### Study design

We sampled at 4 construction sites located in the Copenhagen metropolitan area. The sites were identified by a Confederation of Danish Industry senior consultant to represent larger city construction activities. Three of the sites involved hydraulic drilling rigs, in addition to other subsidiary diesel-powered machines, including excavators, backhoes, loaders, and dump trucks. The fourth site involved a team of excavator operators and ground workers digging a trench and laying a drainage pipe with no drilling rig present. In each case, we sampled over 3 d (Tuesday to Thursday to ensure maximum alignment with the normal workweek of companies, which oftentimes do not work Fridays), from about 0730 to 1600.

### Instrumentation

Scanning mobility particle sizers (Nanoscan SMPS, model 3910, TSI Inc., Shoreview, MN, USA) were used to determine particle number, size distributions, and derived mass-concentrations for particles in the size range of 10 to 420 nm, at time intervals of 1 min, with default particle density set to 1.2 g/cm^3^. Optical particle sizers (OPS, model 3330, TSI Inc., Shoreview, MN, USA) were used to determine particle number, size distributions, and derived mass concentrations for particles in the PM_4_ fraction, at time intervals of 1 min, with default particle density set to 1.0 g/cm^3^. Conductive silicone tubing (TSI Inc., Shoreview, MN, USA) was used to connect SMPSs and OPSs to inlet locations. Portable diffusion size classifiers (DISCMINI, Testo SE & Co. KGaA, Lenzkirch, Germany) were used to determine the total particle number and mean particle diameter for particles in the size range of ~10 to 700 nm, at time intervals of one second. Micro-aethalometers (MA200, AethLabs, San Francisco, CA, USA) fitted with 2.5 µm impactors were used to determine real-time information about BC concentration (as detected in the IR channel of the instrument). 25-mm OD quartz-fiber filters (Whatman QMA) installed in 3-piece cassettes and connected to constant-flow pumps (Apex2, Casella Inc., Bedford, UK) sampling at a rate of 1.9 L air/min were used to quantify EC and OC in the total PM fraction (via thermal and optical properties) in accordance with the EUSAAR2 protocol (Cavalli et al. 2009). Portable data loggers (TinyTag, Gemini TGP-4204 Data Logger) were used to collect real-time temperature and humidity.

### Sampling strategies

Personal measurements were collected from drilling-rig operators situated in cabins (DO), non-drilling machine operators situated in cabins (MO), and ground workers (GW), defined as laborers or machine operators not inside cabins. Stationary measurements were collected at near-field (NF) and far-field (FF) positions. Personal measurements for DO and MO were carried out by installing machine cabins with DISCMINIs and constant-flow pumps attached to quartz-fiber filter cassettes. The sampling inlets were positioned at the operator’s breathing zone (BZ) (Supplementary **Fig. S1A****–****B**). Personal measurements for GW were conducted by equipping their belts or backpacks with constant-flow pumps and a DISCMINI when a backpack was worn (Supplementary **Fig. S1C**). Sampling inlets were attached to their shoulder to sample from their BZ. Stationary measurements were carried out by positioning NF and FF boxes about 3 to 20 m and 20 to 50 m from the target sources, respectively. Each box contained an SMPS, OPS, MA200, DISCMINI, constant-flow pump attached to a quartz-fiber filter cassette, and a temperature and humidity data logger. Inlets were positioned one meter above ground level. At each site, 2 quartz-fiber filter cassettes were deployed as field blanks. Instrument flows were recorded, and constant-flow pumps calibrated before and after each sampling day.

### Sampling sites

**Fig. 1.**
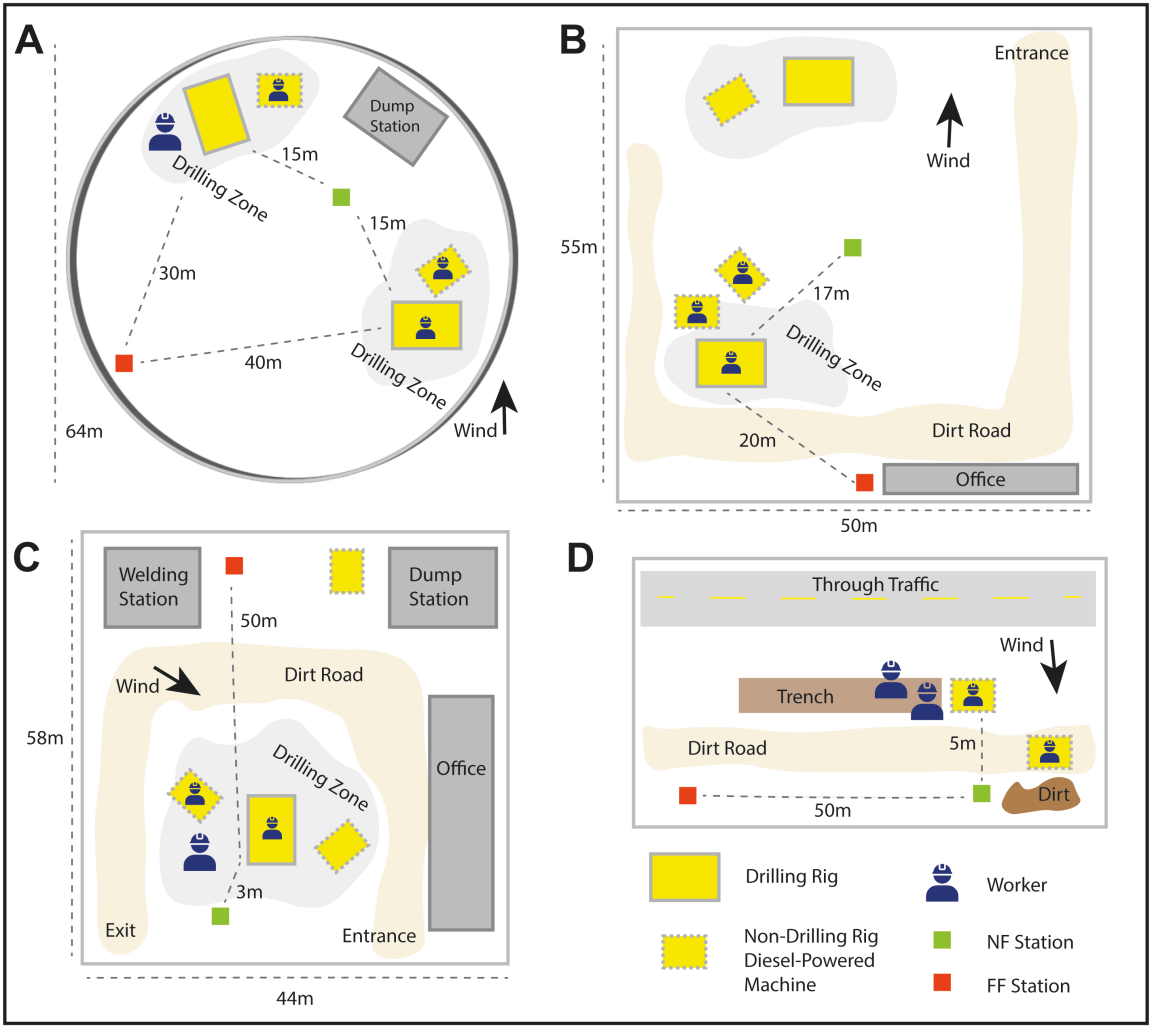
Schematic illustrations of Sites A–D. A legend on the bottom right explains the meaning of the symbols. The blue worker symbol indicates that personal measurements were made here. Site A sits at the bottom of a large cylindrical crater (a future underground parking garage), 64 meters wide and 15 m deep. The drilling zones (light gray) in A–C refer to the ranges over which the drilling rigs moved and operated. The dump stations in A and C were locations where excavators or dump trucks deposited waste materials. The welding station in C housed the workshop of a welding professional. Distances in meters from the near-field (NF) and far-field (FF) stations to the target sources are indicated by dotted lines. Average wind direction (Supplementary **Table S1**) is indicated with an arrow. The entrance and exit labels in B and C lead out to urban streets.

Site A involved the installation of a pile foundation for an underground parking garage (11 to 13 October 2022). The site sat inside a cylindrical crater, ~64 m wide and 15 m deep (**Fig. 1A**). One or two hydraulic drilling rigs drilled 10-m holes, while 2 other machines provided support and moved materials (**Fig. 1A**). One drilling rig (Volvo ECR 88 Plus) was cabin-operated (Supplementary **Fig. S1D**) and the other (Hutte HBR 608-4) was remote-operated by a GW (Supplementary **Fig. S1C**). A wheeled excavator (Volvo EWR 150E) moved waste materials from the drill sites to a disposal container. A mini excavator (Kobelco SK85MSR-7) routinely passed drill casings to the Hutte hydraulic drilling rig. We positioned our FF box against the furthest wall from target sources, ~30 to 40 m from the drilling activities. We positioned our NF box equidistant (~10 to 15 m) from both sources (**Fig. 1A**). On the third day of sampling, the Volvo drilling rig was decommissioned, and only the Hutte was in operation. Personal measurements were made for the sole GW (who wore a backpack) as well as operators of the Volvo drilling rig, wheeled excavator, and mini excavator.

Site B involved the installation of a pile foundation for an apartment building (15 to 17 November 2022). There were 2 teams of workers on site (**Fig. 1B**). One team used an excavator (Caterpillar 330DL VA) and drilling rig (Hutte HBR 608-4) to pile-drive steel sheets into the ground. We focused on a second team, using a larger drilling rig (Liebherr LB20; 56 tons) to drill a series of 10-m-deep holes, along with a mini excavator (Yanmar Vio80) and backhoe (Hydrema 906G) that were providing support and moving materials. A welding professional also repaired drill bits onsite. Other smaller machines routinely passed through the area. On the first day, we set up the NF box ~5 m from the drilling rig but found that this location did not sample the drill emissions adequately due to wind and other obstructions. Hence, we chose to exclude this data from our analysis. On the second and third days, we positioned the NF box instead ~17 m downwind from the drilling rig, nearer to the center of the construction site. This was as close as we could safely position the NF box. On all 3 d, we situated the FF box ~20 m upwind from the drilling rig, just outside of the trafficked region of the construction site (**Fig. 1B**). Personal measurements were made for the operators of the drilling rig, mini excavator, and backhoe. There were no designated GWs on this site.

Site C involved drilling and driving piles for a citywide drainage system (29 November to 1 December 2022). There was a hydraulic rotary drilling rig (Liebherr LB36 XL 410; 126 tons) boring down ~15 m at a diameter of 1 m. Raw materials were deposited from the drill casing into a front loader (Liebherr 556) with a large bucket, which moved them to a designated waste repository (**Fig. 1C**). One of the workers provided mostly ground support for the drilling rig, but also operated a miniature excavator (Takeuchi 200 series) and front loader (JCB 407). A welding professional also provided onsite repairs to the drill bits, both from his welding station and in the drilling zone. When the drill reached its target depth, cement trucks arrived (at the entrance) and deposited cement into the cavity. We positioned our NF and FF boxes about 3 and fifty meters away from the drilling activities, respectively. Personal measurements were collected for GWs and the operators of the drilling rig and front loader.

Site D involved laying of drainage pipe for a light-rail train (4 to 6 October 2022). The general workflow here involved one or two diesel-powered excavators digging a trench and moving materials (mostly fill dirt) while one or two GWs provided support alongside the machines and inside the trench (**Fig. 1D**). On the first day, there was only one excavator (Doosan DX 300 LC). On the next 2 d, there were 2 smaller ones, one on treads (Caterpillar 335F equipped with a Diesel Particle Filtration system) and one on wheels (Hydrema MX16). We positioned a FF station ~50 m upwind of the target source and an NF station ~10 m from the target source (**Fig. 1D**). We adjusted locations throughout the day to keep up with the workers’ progress. Dump trucks and other smaller vehicles are routinely passed by the NF and FF stations. Personal measurements were collected from GWs and MOs.

### Data analysis and statistics

Statistical analyses were carried out using Python’s scipy.stats package. Descriptive statistical analysis was used to calculate arithmetic and geometric means, as well as standard deviations. Analysis of variance (ANOVA) was used to determine statistically significant differences across samples. When an ANOVA indicated significant differences, Tukey’s Honestly Significant Difference (HSD) post-hoc test was used to carry out pairwise comparisons. Throughout the paper, “average” refers to the geometric mean (GM) unless otherwise specified.

## Results

### Summary of EC and OC measurements

**Fig. 2.**
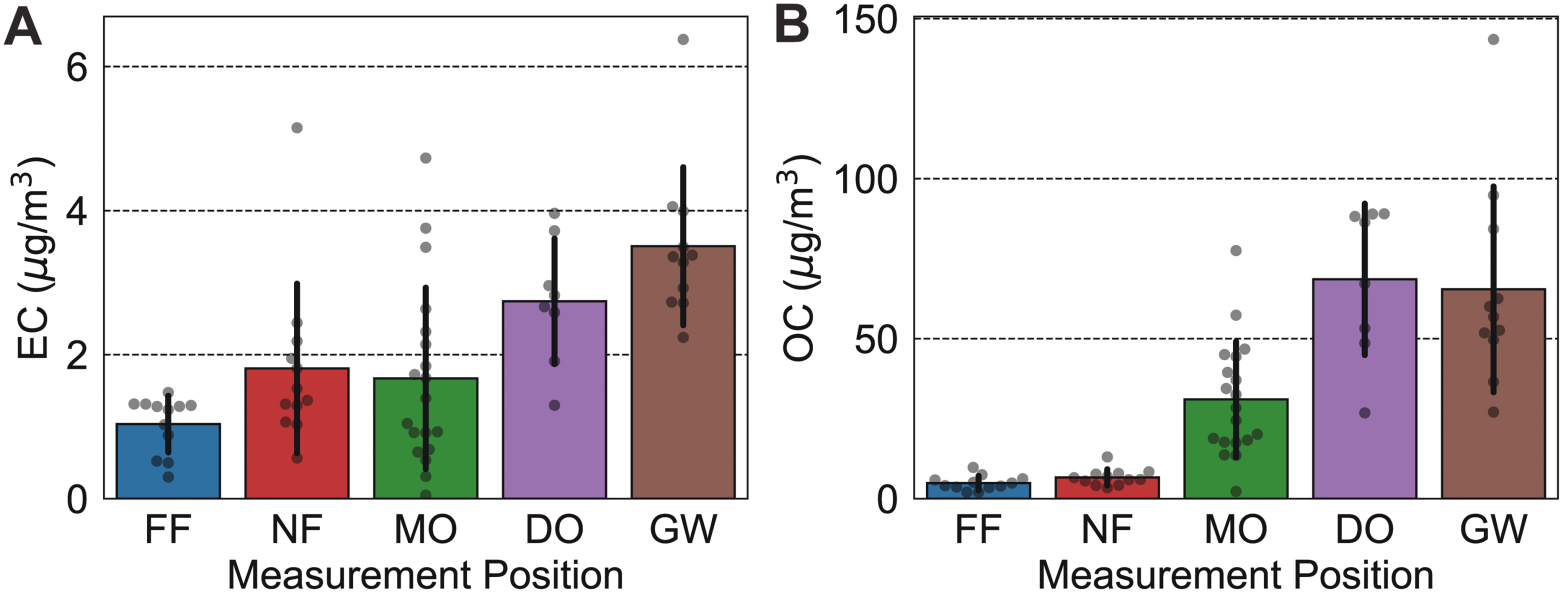
Summary of EC/OC measurements. Arithmetic averages and standard deviations are shown. Each dot represents the (A) elemental carbon or (B) organic carbon concentration measured over one full shift (6 to 8 h) on quartz-fiber filters positioned on ground workers (GW), drilling rig operators (DO), or non-drilling machine operators (MO), as well as at near-field (NF) or far-field (FF) stations. EC measurements for DO (*P* = 0.007) and GW (*P* < 0.0005) differed significantly from FF. EC measurements for GW also differed significantly from MO (*P* = 0.0002) and NF (*P* = 0.005). OC measurements for MO (*P* = 0.004), DO (*P* < 0.0005), and GW (*P* < 0.0005) differed significantly from FF.

In total, we collected 61 full-shift EC/OC samples from 16 workers (5 GW, 3 DO, and 8 MO) and 8 stationary positions (4 NF and 4 FF). **Table 1** summarizes measurements made at each position and includes arithmetic and geometric mean (AM and GM) values, as well as hours sampled. Across the 4 sites, the average background (FF position) concentration was 0.9 µg/m^3^ EC (**Fig. 2A**, **Table 1**). GW samples had the highest average EC concentration (3.4 µg/m^3^), followed by DO samples (2.6 µg/m^3^), and MO samples (1.2 µg/m^3^) (**Fig. 2A**, **Table 1**). An ANOVA indicated the presence of a significant difference amongst some of the positions (*P* < 10^−6^) and a post-hoc test (Tukey’s HSD) confirmed that both the DO (*P* = 0.007) and GW (*P* < 0.0005) positions differed significantly from the background. Moreover, the GW position differed significantly from both the MO (*P* = 0.0002) and NF (*P* = 0.005) positions. Overall, the MO position had the widest range in EC concentration: nearly zero to 4.74 µg/m^3^ (**Fig. 2A**). The arithmetic average EC concentration measured for each MO position groups into 2 clusters: 5 from 0.6 to 1.0 µg/m^3^, in line with the FF measurements, and 3 from 2.0 to 4.0 µg/m^3^ (Supplementary **Table S2**). Our NF positions, on average, did not differ significantly from the FF positions (*P* = 0.4), which we explain by the fact that NF stations were located (for safety and practical reasons) at least 3 m from the target emission source, which is far enough for significant levels of dilution of particulate matter. Nonetheless, the NF position at site B, with an arithmetic average EC concentration of 3.8 ± 1.9 µg/m^3^, did exceed background concentrations (Supplementary **Table S2**).

**Table 1.** Summary of online and offline measurements.

Site	Position	EC µg/m^3^	OC µg/m^3^	EC/TC	BC µg/m^3^	TPN-SMPS 10^3^/cm^3^	TPN-DM 10^3^/cm^3^	PM_4_ µg/m^3^
All	NF (*n* = 11) 4 positions	AM = 1.9 ASD = 1.2 GM = 1.6 GSD = 1.8 *t* = 87.7	AM = 6.5 ASD = 2.6 GM = 6.1 GSD = 1.4 *t* = 87.7	0.23	AM = 2.3 ASD = 4.1 GM = 0.65 GSD = 8.0 *t* = 86.2	AM = 24.3 ASD = 66.4 GM = 10.6 GSD = 3.0 *t* = 83.6	AM = 18.6 ASD = 67.0 GM = 7.8 GSD = 2.9 *t* = 45.9	AM = 17.4 ASD = 28.7 GM = 12.2 GSD = 2.0 *t* = 83.6
All	FF (*n* = 12) 4 positions	AM = 1.0 ASD = 0.4 GM = 0.9 GSD = 1.7 *t* = 96.5	AM = 4.9 ASD = 2.2 GM = 4.4 GSD = 1.6 *t* = 96.5	0.17	AM = 0.80 ASD = 1.3 GM = 0.26 GSD = 8.4 *t* = 93.8	AM = 10.8 ASD = 21.8 GM = 6.0 GSD = 2.8 *t* = 93.0	AM = 10.3 ASD = 20.6 GM = 6.4 GSD = 2.3 *t* = 56.0	AM = 10.2 ASD = 13.1 GM = 8.0 GSD = 1.9 *t* = 86.1
All	GW (*n* = 11) 5 workers	AM = 3.5 ASD = 1.1 GM = 3.4 GSD = 1.3 *t* = 77.9	AM = 65 ASD = 32 GM = 59 GSD = 1.6 *t* = 77.9	0.05	NA	NA	AM = 14.5 ASD = 53.8 GM = 7.7 GSD = 2.9 *t* = 17.3 *only 1 worker*	NA
All	DO (*n* = 8) 3 workers	AM = 2.7 ASD = 0.9 GM = 2.6 GSD = 1.4 *t* = 66.0	AM = 69 ASD = 24 GM = 64 GSD = 1.5 *t* = 66.0	0.04	NA	NA	AM = 44 ASD = 117 GM = 12.3 GSD = 4.2 *t* = 42.3	NA
All	MO (*n* = 19) 8 workers	AM = 1.7 ASD = 1.3 GM = 1.2 GSD = 2.9 *t* = 145.6	AM = 31 ASD = 18 GM = 25 GSD = 2.1 *t* = 145.6	0.05	NA	NA	AM = 35 ASD = 238 GM = 6.5 GSD = 3.6 *t* = 84.0	NA
Drilling sites	NF (*n* = 8) 3 positions	-	-	-	AM = 2.5 ASD = 4.3 GM = 0.70 GSD = 8.3 *t* = 62.9	AM = 31.9 ASD = 77.2 GM = 14.8 GSD = 2.9 *t* = 59.8	AM = 24.7 ASD = 79.1 GM = 10.2 GSD = 3.2 *t* = 30.6	AM = 21.1 ASD = 32.9 GM = 15.2 GSD = 2.0 *t* = 59.8
Drilling sites	FF (*n* = 9) 3 positions	-	-	-	AM = 0.9 ASD = 1.5 GM = 0.3 GSD = 6.7 *t* = 70.0	AM = 12.5 ASD = 24.9 GM = 6.6 GSD = 3.1 *t* = 68.8	AM = 12.7 ASD = 24.5 GM = 7.4 GSD = 2.6 *t* = 35.6	AM = 10.0 ASD = 12.1 GM = 8.0 GSD = 2.0 *t* = 61.9

EC, elemental carbon; OC, organic carbon; TC, total carbon; BC, black carbon; TPN-SMPS, total particle number measured via SMPS; TPN-DM, total particle number measured via DISCMINI; NF, near-field; FF, far-field; GW, ground worker; MO, non-drill-rig machine operator; DO, drilling rig operator; AM, arithmetic mean; ASD, arithmetic standard deviation; GM, geometric mean; GSD, geometric standard deviation; *n*, number of days sampled; *t*, total time in hours; NA, not applicable.

Personal OC measurements showed much higher levels than at the stationary positions (**Fig. 2B**, **Table 1**). The DO samples had the highest average OC concentration (64 µg/m^3^), followed by the GW samples (59 µg/m^3^), and MO samples (25 µg/m^3^) (**Fig. 2B**, **Table 1**). NF and FF samples showed far lower concentrations of OC, 6.1 and 4.4 µg/m^3^, respectively. Post-hoc testing (Tukey’s HSD) confirmed that the DO (*P* < 0.0005), MO (*P* = 0.004), and GW (*P* < 0.0005) samples were significantly higher in concentration compared to the FF position (equally true for the NF position). Moreover, DO and GW samples were significantly higher than the average MO sample.

### Summary of real-time measurements

**Fig. 3.**
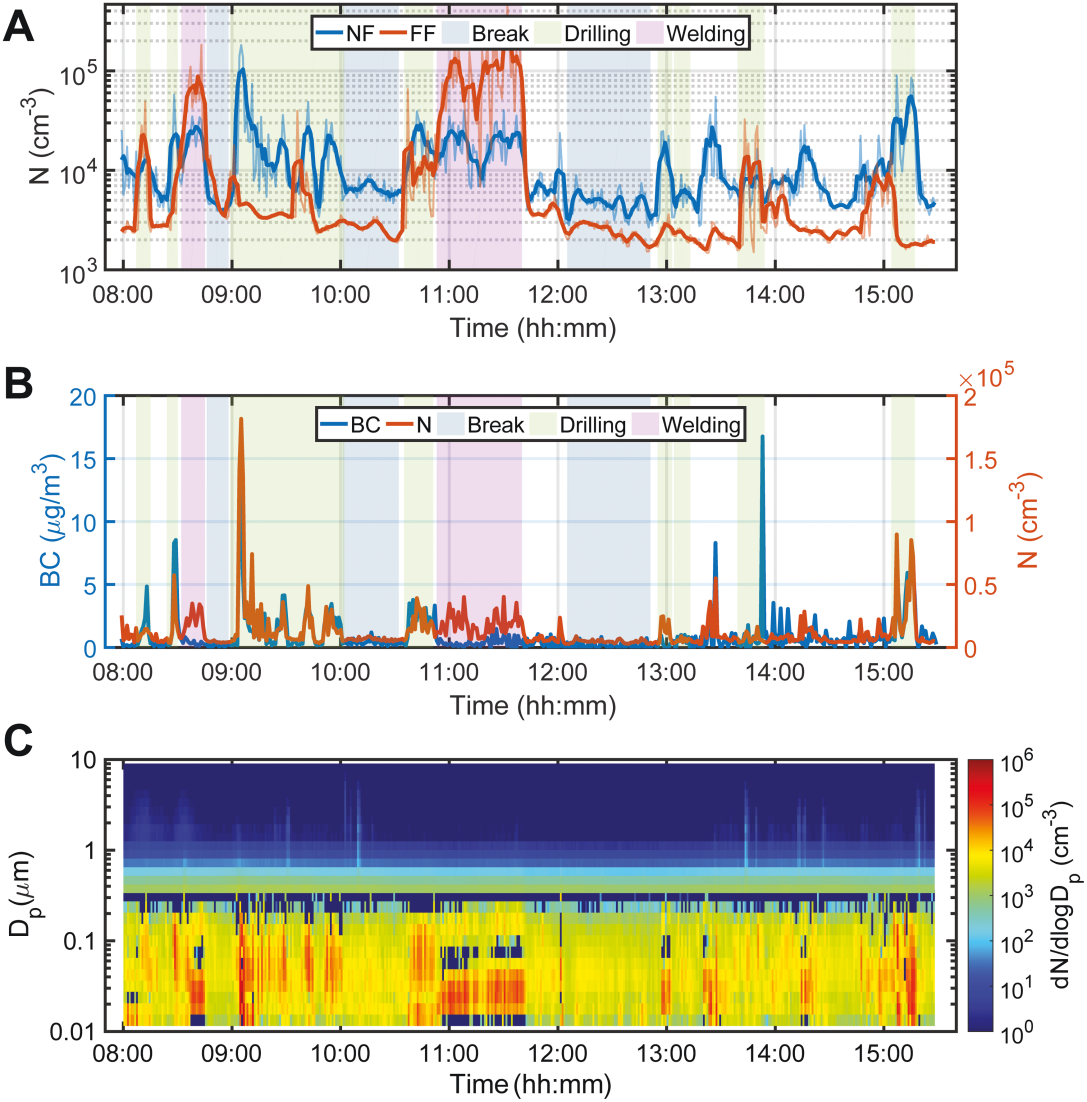
Exemplary time-course plots of particle number and black carbon concentrations. (A) Time-course plot of total PNC measured via SMPS at the NF and FF stations on day 3 of sampling at site C. Darker lines reflect 5-min moving means. Highlights indicate activity status (Break, Drilling, or Welding). (B) Time-course plot of black carbon concentration at the NF station on the left axis (blue) and PNC on the right axis (orange). Highlights indicate activity status (Break, Drilling, or Welding). (C) A contour plot, generated from a composite of SMPS and OPS data sets, showing the particle size distribution over time at the NF station.

**Figure 3** shows representative real-time measurements of total PNC at NF and FF stations (**Fig. 3A**), BC concentration compared to PNC at the NF station (**Fig. 3B**), and particle size distribution (PSD) over time at the NF station (**Fig. 3C**). A complete collection of PSD contour plots is included in Supplementary **Fig. S5**.

Over the 4 sites, the average total PNCs determined via SMPS and OPS in the NF and FF positions were about 10,600 (GSD = 3.0) and 6,000 (GSD = 2.8)/cm^3^, respectively (**Table 1**). This was largely consistent with our measurements made via DISCMINI, about 7,800 (GSD = 2.9) and 6,400 (GSD = 2.3)/cm^3^ at the NF and FF stations, respectively (**Table 1**). It is important to mention that the DISCMINIs generally sampled for less time (45.9 h) than their SMPS counterparts (83.6 h) (**Table 1**), which may explain some of the discrepancy. When we consider only sites with drilling rigs, the average total PNC at NF positions shifts up to 14,800/cm^3^ (GSD = 2.8) with the SMPS/OPS and 10,200/cm^3^ (GSD = 3.2) with the DISCMINI (**Table 1**). Site B saw the highest overall particle numbers: 32,000/cm^3^ (GSD = 3.4) at the NF station and 4,200/cm^3^ (GSD = 2.1) at the FF station (Supplementary **Table S2**). The maximum total PNC reached on site B was 230,000/cm^3^ (see Supplementary **Fig. S4** for those dynamics). Based on personal DISCMINI measurements, GWs, DOs, and MOs were exposed to average total PNCs of 7,700 (GSD = 2.9), 12,300 (GSD = 4.2), and 6,500 (GSD = 3.6)/cm^3^, respectively (**Table 1**). In terms of particulate mass, the overall average calculated concentrations of PM_4_ (particulate matter with an aerodynamic spherical equivalent d_50_ = 4 µm) in the NF and FF positions were about 12.2 (GSD = 2.0) and 8.0 (GSD = 1.9) µg/m^3^, respectively (**Table 1**).

The overall average BC at the NF and FF stations was 0.65 (GSD = 8.0) and 0.26 (GSD = 8.4) µg/m^3^ BC, respectively, but as the GSDs indicate, the range was wide (**Table 1**). On site B, the maximum concentration was 44 µg/m^3^ BC, and the maximum 15-min moving mean was 17 µg/m^3^ BC (see Supplementary **Fig. S4**).

### Summary of activity-based real-time measurements

**Fig. 4.**
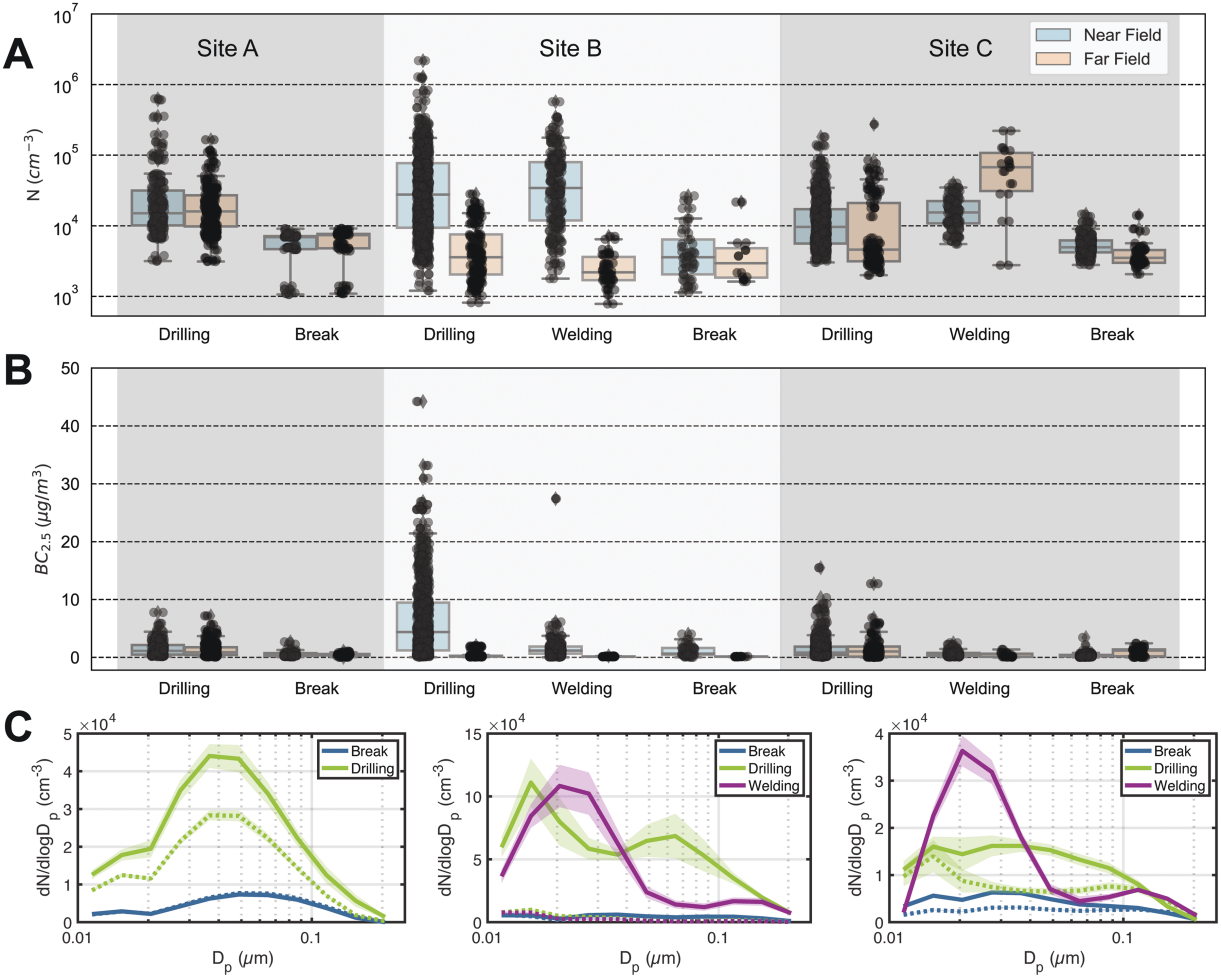
Cross-study comparison of particle number concentration, black carbon concentration, and particle size distribution. (A) Total particle number concentration measured via SMPS at the NF and FF stations during drilling, welding, and breaks on sites A–C. (B) Black carbon concentration measured via aethalometer at the NF and FF stations during drilling, welding, and breaks on sites A-C. (C) Average particle size distributions for sites A-C.

We categorized workplace activities as drilling, breaks, or welding (**Fig. 3A–B**). Drilling was associated with significantly elevated total PNCs compared to breaks (**Figure 4A**). On sites A-C, the average total PNCs measured via SMPS and OPS at NF stations during drilling were about 20,800, 35,000, and 11,100/cm^3^, respectively, compared to 4,600, 4,100, and 5,100/cm^3^ during pauses (Supplementary **Table S2**). On sites B and C, welding similarly showed elevations in average total PNCs, about 30,000 and 15,600/cm^3^, respectively (Supplementary **Table S2**). Drilling was also associated with elevated BC production. On sites A-C, the average concentrations measured at the NF station during drilling were 1.0, 4.2, and 0.67 µg/m^3^ BC, respectively, compared to 0.35, 0.13, and 0.11 µg/m^3^ BC measured during the pauses (Supplementary **Table S2**). As expected, welding on sites B and C was not associated with elevated levels of BC, about 0.66 and 0.28 µg/m^3^ at the NF stations, respectively (Supplementary **Table S2**). Particle emissions from drilling were mostly in the ultrafine size range, as is evident in the contour plots (**Fig. 3C**, Supplementary **Fig. S5**), which show increased emission of particles smaller than about 100 nm during drilling. This observation is made more evident in the average PSD plots (**Fig. 4C**). The main particle-size modes observed during drilling included one at ~40 nm on site A; one at ~15 nm and another at 60-70 nm on site B; and one at ~30 nm on site C (**Fig. 4C**). Welding had a particle size mode of ~20 nm on both sites B and C (**Fig. 4C**).

We also checked for correlations between particle number and BC concentrations during drilling. Although we see a weak positive correlation (*r* = 0.23) overall, we find a much stronger positive correlation (*r* = 0.53) when we focus only on drilling at site C (Supplementary **Fig. S6**). This is assumed to be due to the closer proximity of the NF station to the target source at site C than at the other sites (**Fig. 1C**).

## Discussion

We investigated workplace exposures to DEE in terms of EC and PNC on large outdoor construction sites. Background levels of DEE (0.9 µg/m^3^ EC) were consistent with recent measurements made on a busy street corner in Copenhagen (0.59 µg/m^3^ EC) (Ellermann et al. 2022a,  2022b). Personal exposures for GWs (3.4 µg/m^3^ EC) and DOs (2.6 µg/m^3^ EC) were significantly higher than the background, but samples from MOs (1.2 µg/m^3^ EC) were not (**Table 1**).

These concentrations might be considered relatively low personal EC exposures. In the extreme case of miners, for instance, it was common in the past to see workday exposures above 100 or even 500 µg/m^3^ EC (Pronk, Coble, and Stewart 2009), though in recent years these numbers have come down significantly (7 µg/m^3^ EC) (Gren et al. 2022). In construction, tunnel workers generally have the highest levels of DEE exposure. In an influential survey of occupations in Sweden, tunnel workers showed significantly higher levels of exposure (86.7 µg/m^3^ EC) than their counterpart diesel-powered machine operators on construction sites (7.8 µg/m^3^ EC) (Lewné et al. 2007). Though, like the miners, these levels of exposure have been decreasing, as indicated in another more recent study of Swedish tunnel workers, which reported an exposure (2.6 µg/m^3^ EC) similar to what we see for DOs (Hedmer et al. 2017).

A recent study of Canadian construction workers reported that outdoor workers were exposed to much lower levels (1.5 µg/m^3^ EC) than underground workers (11.2 µg/m^3^ EC) (Ziembicki et al. 2022). They also found that enclosed cabins on heavy equipment reduced DEE exposure by >50%, an observation supported elsewhere in the literature (Park et al. 2020), and one that might explain the relatively wide range of exposure for non-drilling-rig machine operators in this study. Whereas the DOs in our study consistently kept their cabin doors open (exposed to the surrounding environment), other machine operators were much more variable. Though not construction, another recent study investigated DEE exposure in American farm workers at harvest (Stapleton et al. 2018). Like construction workers, farm workers work outdoors and commonly operate diesel-powered machines. Indeed, their levels of exposure (0.1 to 2.7 µg/m^3^ EC) were quite comparable to those reported here. In summary, outdoor construction-like work is associated with average workday levels of DEE exposure less than about 5 µg/m^3^ EC. Although this is below the Danish occupational exposure limit (10 µg/m^3^) and well below the European limit (50 µg/m^3^), there is 1:1,000 cancer risk at 0.45 µg EC /m^3^ (Saber et al. 2018). Consequently, suggestions have been circulating that the EC OEL should be much lower (Taxell and Santonen 2016; Keefe et al. 2020).

Discrepancies observed in stationary and personal OC exposure are likely due to cigarette smoking, which was noted frequently in the logbooks. Cigarette smoke is a generous source of OC content (Hildemann et al. 1991). Therefore, it is not surprising to see much higher OC in our personal measurements. Supplementary **Figure S2** illustrates an inverse relationship between the ratio of EC to total carbon (TC) against OC. Whereas NF and FF measurements have EC/TC ratios comparable to the urban background (Ellermann et al. 2022a), the personal measurements are far lower, which is in agreement with our assumption of OC contribution from cigarette smoking. If the OC originated from the diesel engines, we would expect much higher EC/TC ratios (Maricq 2007). Furthermore, cigarette smoke should not confound our evaluation of DEE exposure as cigarette smoke does not produce significant levels of EC content (Hildemann et al. 1991; Schauer 2003). In fact, OC/EC ratios as high as 57 have been reported in homes with frequent indoor smokers (Na and Cocker 2005).

Occupational DEE exposure is typically measured using a NIOSH 5040 protocol. In such cases, it is not unusual for EC concentration to surpass ambient levels by orders of magnitude, as illustrated in underground mines (Pronk et al. 2009). The NIOSH 5040 protocol applies higher temperatures in the final He/O_2_ mode, which burns elevated concentrations of refractive EC (Cavalli et al. 2009). However, it also applies higher temperatures in the preceding He mode, which may cause premature evolution of refractive carbon and thereby underestimate EC (Cavalli et al. 2009; Chiappini et al. 2014; Bautista et al. 2015). Higher temperatures in the He mode may also cause more charring of OC, which contributes to the uncertainty of pyrolytic carbon and, thereby, the split point of OC/EC. The EUSAAR-2 protocol (the one we use here) was developed to minimize this charring in background aerosols, where EC concentrations are typically lower than in the occupational environment. Furthermore, since ambient aerosol filter samples in Europe are typically analyzed using the EUSAAR-2 protocol, occupational exposures can be directly compared to ambient levels at various locations. In order to check if the lower temperature in the He/O_2_ mode caused incomplete evolution of EC in filters with higher loading of EC, we plotted the residual laser transmittance against ambient EC concentration. We found no correlation (*r*^2^ = 0.02) and concluded that incomplete evolution of refractive EC using the EUSAAR-2 protocol was not a problem at the observed concentrations.

Calculated PM mass concentrations were relatively low, especially when compared to tasks like concrete mixing (Vinnikov et al. 2023) or demolition (Bello et al. 2019). Whereas the Danish OEL for respirable nontoxic dust is 5,000 µg/m^3^, the overall average on drilling-rig sites was orders of magnitude lower than that (15.2 µg/m^3^) (**Table 1**). This is consistent with the fact that the majority of DEE particles are in the fine (<300 nm) and ultrafine (<100 nm) size ranges and therefore do not contribute much to mass (Ris 2007). Given that the main modes in particle diameter during the drilling activities were always less than 100 nm (**Fig. 4C**), we argue that it is more sensible to focus on PNCs as indicators of DEE and the observed welding activities. Moreover, environmental exposure to combustion-derived ultrafine particles, of which DEE is a substantial contributor, has long been associated with a wide suite of health effects such as pulmonary inflammation, asthma, neurotoxicity, and progression of cardiovascular disease, besides cancer (Costa et al. 2014; Stone et al. 2017). Epidemiological studies suggest that an increase in ultrafine particle concentrations may relate to some acute to subacute effects (Gardner et al. 2014; Stafoggia et al. 2017; Fang et al. 2022). Average total PNCs varied significantly across the worksites, but sites with active drilling rigs were consistently higher (13,600, 32,000, and 9,700 versus 4,700/cm^3^) (Supplementary **Table S2**). Average total PNCs measured during drilling varied from 11,000 to 35,000/cm^3^ (**Fig. 4A**). In comparison, welding and other metal-work activities are reported to produce the highest workplace concentrations of ultrafine particles (~10^4^ to 10^6^/cm^3^) (Viitanen et al. 2017). Most of our measurements were on the lower end of that spectrum, similar to those reported for a truck workshop in Canada (22,700/cm^3^) (da Silveira Fleck et al. 2020) or during traffic in Copenhagen (15,000/cm^3^) (Ellermann et al. 2022b).

This study makes clear how much personal exposure measurements can differ from NF stations, even when NF stations are only a few meters away from the target source. Regarding both DEE and PM, we saw that the NF stations showed significantly lower levels, and in fact, EC concentrations did not differ significantly between the NF and FF stations. This is not so surprising in the cases where the NF station was over 10 meters from the source (plenty of room for dilution). But at site C, for instance, the NF station (AM = 1.8 µg/m^3^ EC; 25.6 h) was only 3 m from the drilling location, yet the GW position (AM = 4.6 µg/m^3^ EC; 24.6 h) showed much more DEE exposure. Still, these results from NF and FF stations as well as GWs are indicative of bystander exposure at such work sites.

The relatively wide variation in measured exhaust pollutants across the 4 sites might be due to engine-emission classifications and engine loads, as well as fuel type and quantity used per day. EU regulation ranks engines on a scale of I to VI (EC 2011; Transport Policy 2018), with class VI similar to 2010 United States standards. The engines in this study ranged from ECII to ECV (Supplementary **Table S3**). The largest drilling rig by engine load (390 kW/ 523 HP) was on site C, which also showed the lowest particle concentrations of the 3 drilling sites, even though it was also the closest to the NF station (**Fig. 1**). We suggest that this may be explained by the fact that that particular drilling rig was rated ECIV, which requires more stringent emission restrictions than the ECIIIA rig on site B (270 kW/ 362 HP), which was associated with the highest DEE and particle concentrations. Moreover, although site A had a drilling rig with the lowest classification (ECII), this rig was also the smallest in the study (38.2 kW/ 52 HP). In terms of fuel, all machines burned 7% biodiesel, but their average daily intake varied from 5 to 11 liters/h (for smaller excavators) up to 25 to 90 liters/h for the drilling rig on site C (Supplementary **Table S3**). Nevertheless, information on actual daily fuel use was not collected, making a more detailed analysis unfeasible.

Some reports suggest that DEE exposures increase in colder environments (Ziembicki et al. 2022). Though we sampled over a relatively short time window (October and November), this also coincided with significant changes in temperature. Whereas site D had much more moderate weather (AM = 16.2 ± 0.4 °C), the temperature had dropped significantly by the time we reached site C (AM = 4.8 ± 0.9 °C) (Supplementary **Table S1**). We do not have enough data to comment further on the impact of temperature.

One of the main limitations of this study is the measurement on a relatively small number of workers in each scenario. For instance, although we sampled on 8 d at the DO position, these measurements were only made on 3 different drilling rigs. Another limitation is that for safety and practical reasons, it was often difficult to situate the NF stations closer to the target sources, so particle number and BC concentrations from NF stations are indicative of the general environment on-site but suitable for identifying the DEE activities. Moreover, the distance between NF stations and target activities ranged from 3-17 meters (**Fig. 1**), a fact that needs to be taken into consideration when drawing any comparisons with reported concentrations. Last, due to a limited availability of instruments, we only measured BC at stationary positions. Hence, although we were able to report correlations between BC and ultrafine particle concentrations at stationary positions, we were unable to use this data to draw conclusions about personal exposure to BC throughout the workday.

## Conclusion

DEE exposures amongst outdoor construction workers were below European (50 µg/m^3^ EC) and Danish (10 µg/m^3^ EC) OELs. Samples from GWs (3.4 µg/m^3^ EC) and DOs (2.6 µg/m^3^ EC) were significantly higher than background, but samples from MOs (1.2 µg/m^3^ EC) were not. Real-time sampling of BC concentration showed that there are many acute bursts of DEE exposure (often above 5 and some as high as 44 µg/m^3^ BC), which may be a matter of concern beyond the 8-h TWA exposure limits. This is also true of ultrafine particles, which are not currently regulated, but are a health concern and, as our study shows, make a serious contribution to occupational exposures near diesel-powered machines.

## Supplementary material

Supplementary material is available at *Annals of Work Exposures and Health* online.

wxae062_suppl_Supplementary_Figures_S1-S6_Tables_S1-S3

## Data Availability

Data and software (MATLAB and Python scripts) used throughout this project are held by the corresponding author and available upon request.
