# Supplementary material for: Airborne particulate matter and diesel engine exhaust on infrastructure construction sites in the Copenhagen metropolitan area
Source: Ann Work Expo Health. 2024 Sep 17;68(8):791–803. doi: 10.1093/annweh/wxae062 (PMC11427543; doi:10.1093/annweh/wxae062)
Supplement: wxae062_suppl_Supplementary_Figures_S1-S6_Tables_S1-S3 [file wxae062_suppl_supplementary_figures_s1-s6_tables_s1-s3.pdf]

Airborne particulate matter and diesel engine exhaust on infrastructure construction sites in the Copenhagen metropolitan area

Patrick L. Ferree, Merve Polat, Jakob K. Nøjgaard, Keld A. Jensen*

The National Research Centre for the Working Environment, Lersø Parkallé 105, Copenhagen DK-2100, Denmark

*Corresponding author: kaj@nfa.dk

Supplemental Information

This document provides supplemental figures and tables for the main text.

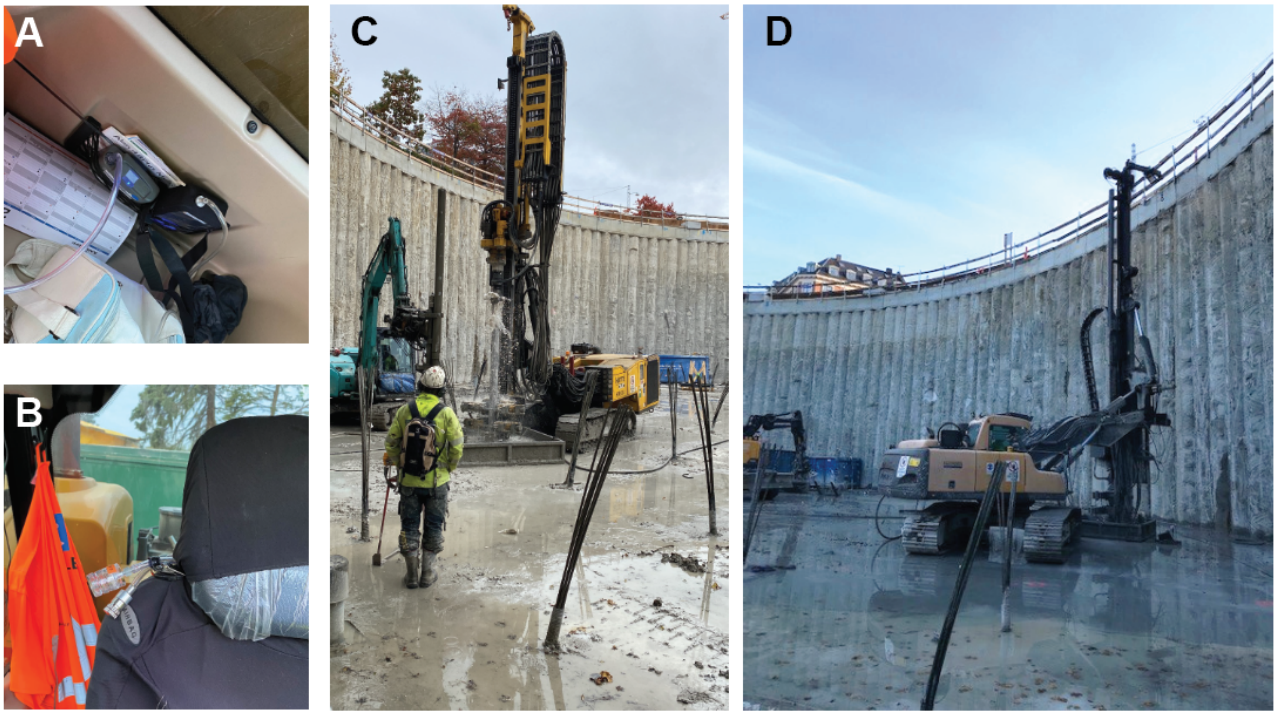

Figure S1. Photographs of representative machines and instrument set-ups. (A) A diffusion size classifier and a constant-flow pump inside the cabin of a drilling rig. (B) Instrument inlets on the headrest of the operator seat. (C) Ground worker wearing portable sampling devices in a backpack while operating a drilling rig at Site A. (D) Cabin-operated drilling rig at Site A.

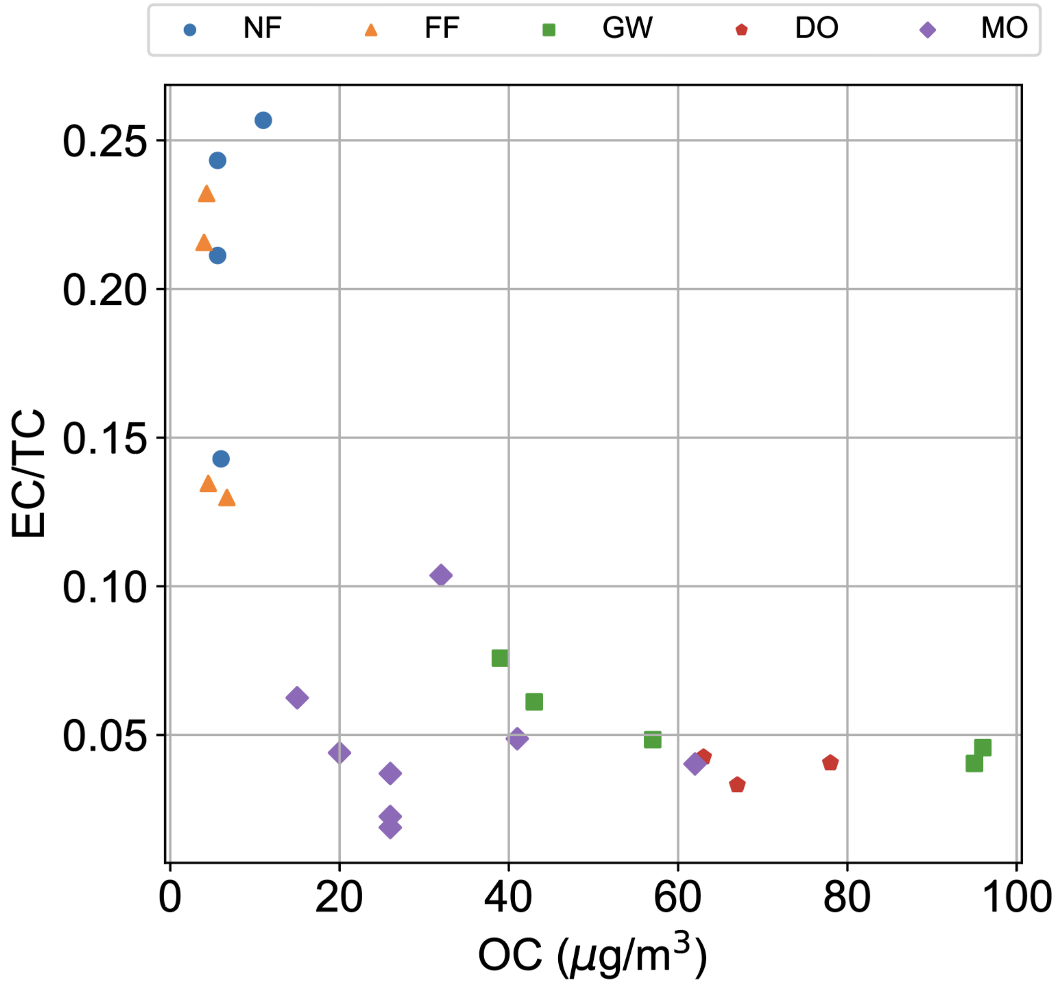

Figure S2. Analysis of source dependency of relative EC. EC/TC ratio plotted against OC concentration. TC (total carbon) is the sum total of elemental and organic carbon. Average EC and OC values were used to make this plot.

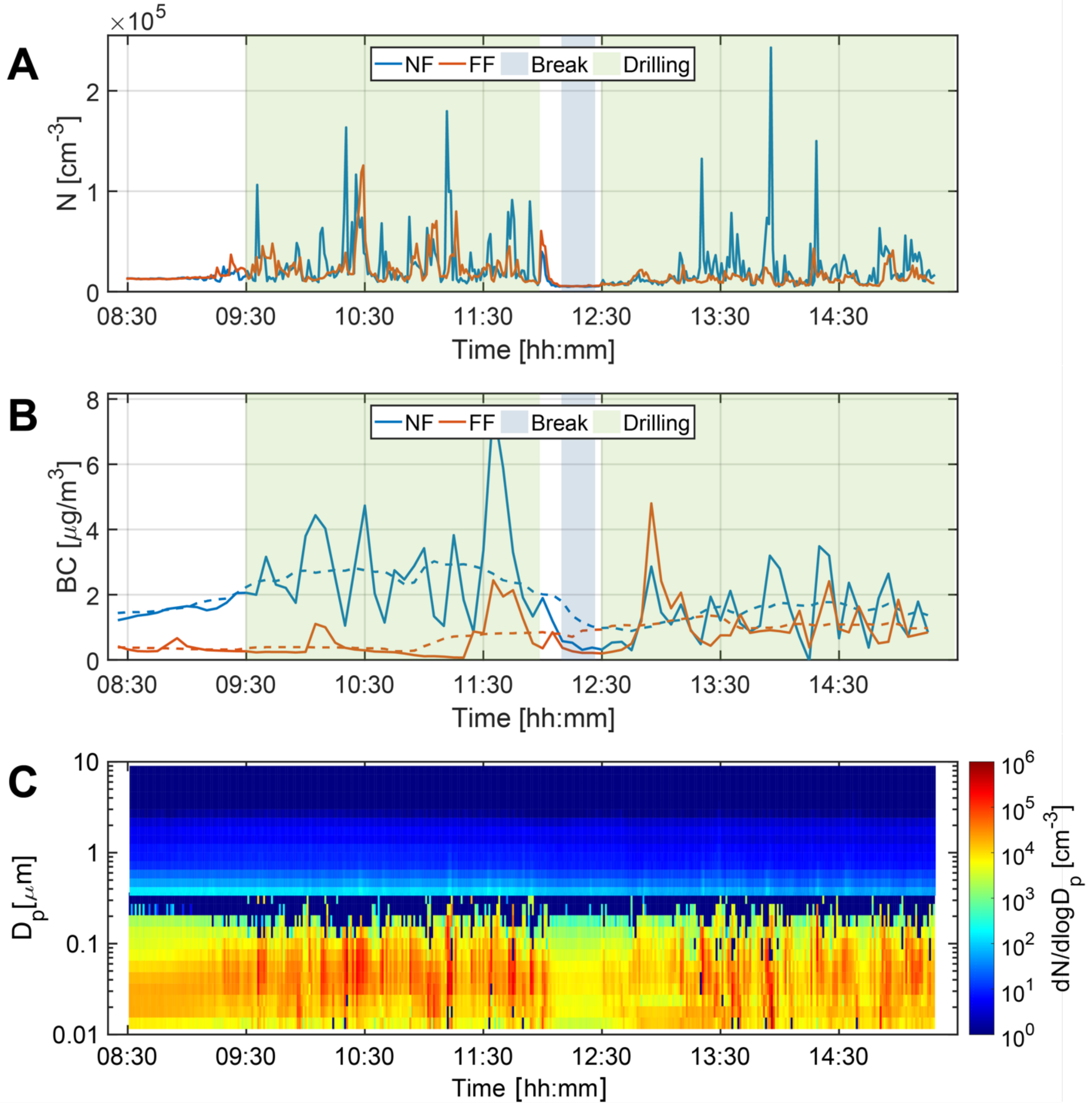

Figure S3. Exemplary time-course plots of particle number and black carbon concentrations on Site A. (A) Time-course plot of total particle number concentration measured via SMPS at the NF and FF stations on day 2 of sampling at site C. Darker lines reflect 5-minute moving means. Highlights indicate activity status. (B) Time-course plot of black carbon concentration at the NF station. Highlights indicate activity status. (C) A contour plot, generated from a composite of scanning mobility and optical particle sizer data sets, showing the particle size distribution over time in the NF station.

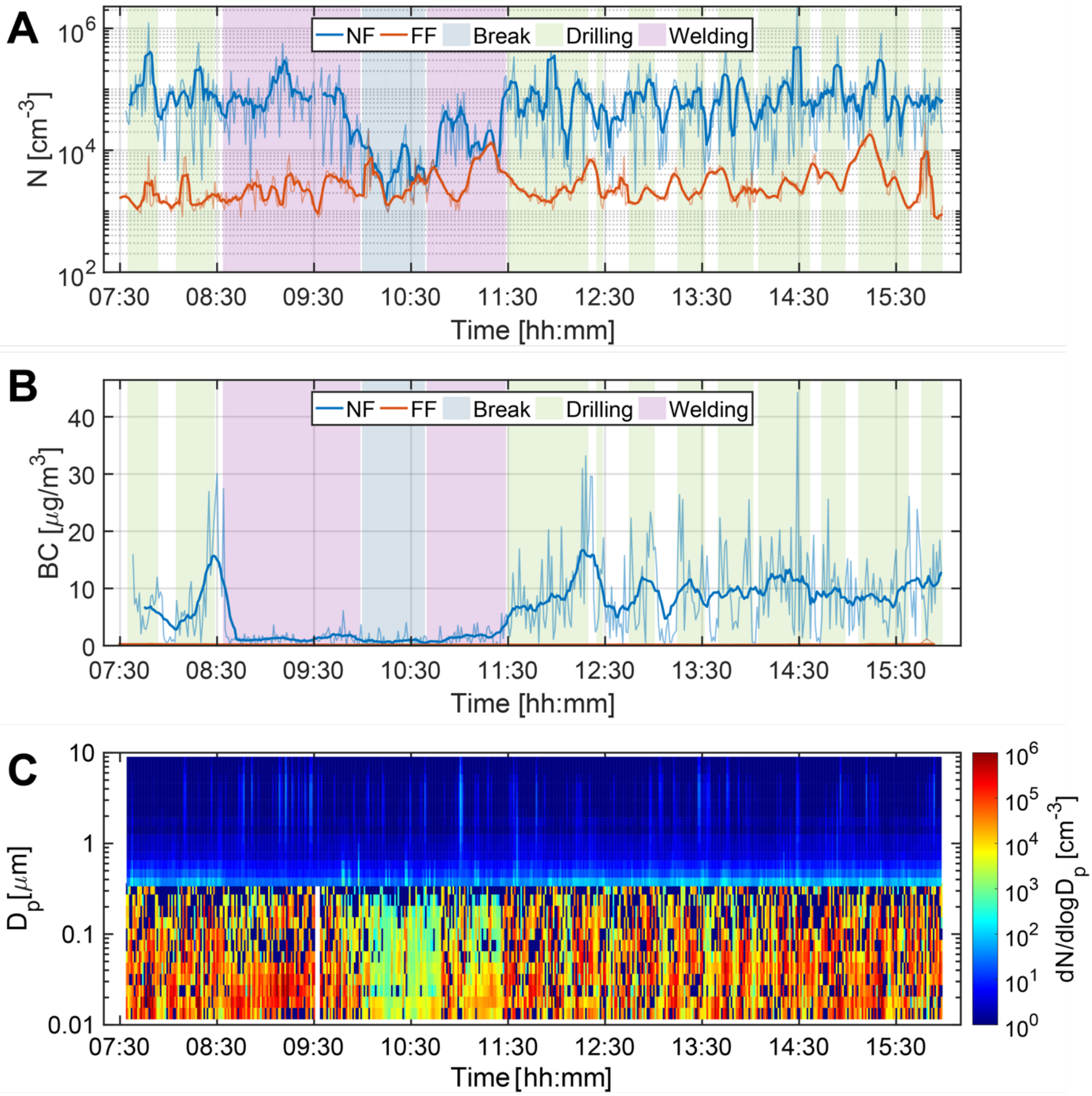

Figure S4. Exemplary time-course plots of particle number and black carbon concentrations on Site B. (A) Time-course plot of total particle number concentration measured via SMPS at the NF and FF stations on day 2 of sampling at site C. Darker lines reflect 5-minute moving means. Highlights indicate activity status. (B) Time-course plot of black carbon concentration at the NF station. Highlights indicate activity status. (C) A contour plot, generated from a composite of scanning mobility and optical particle sizer data sets, showing the particle size distribution over time in the NF station.

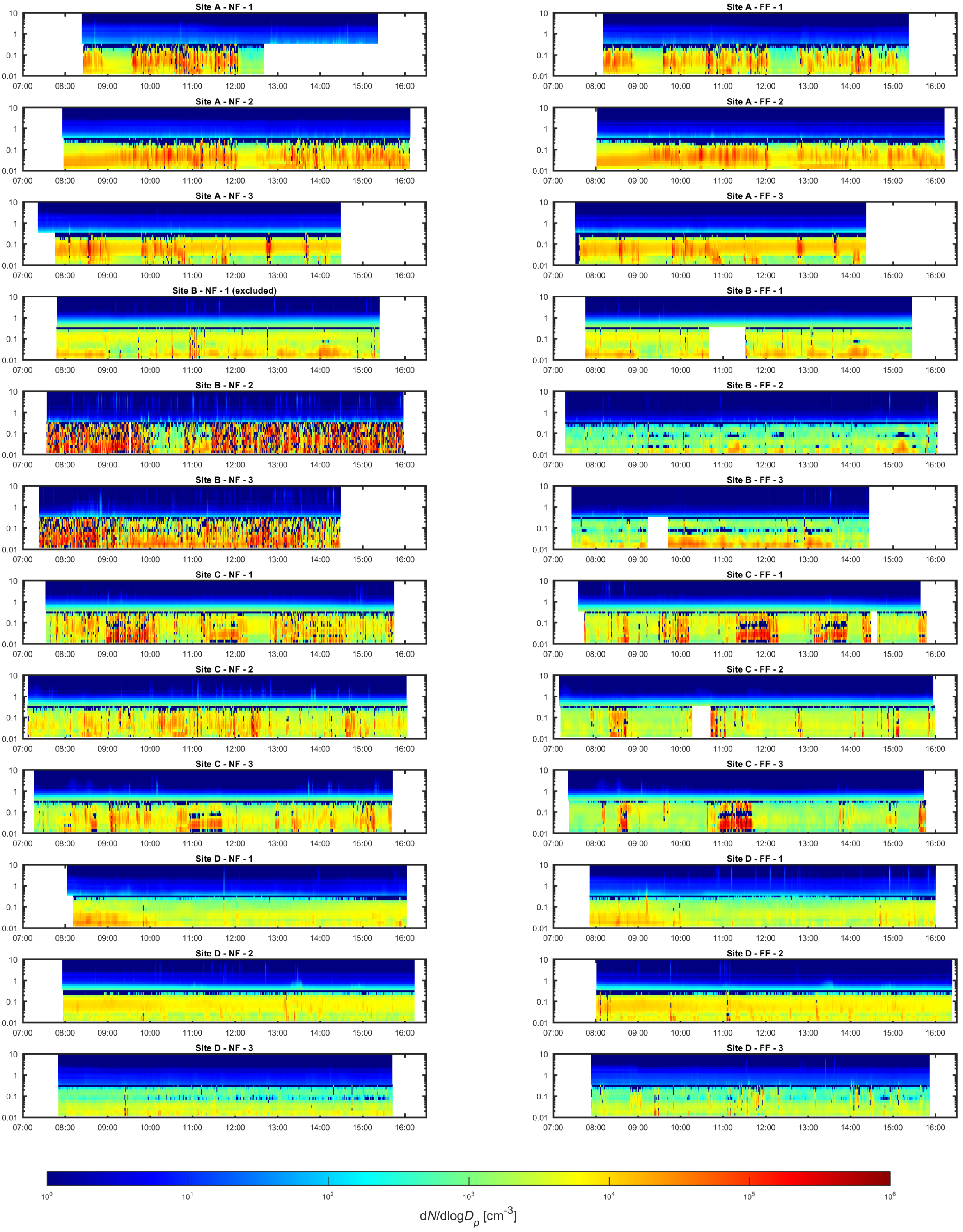
Site A - NF - 1
Site A - FF - 1
Site A - NF - 2
Site A - FF - 2
Site A - NF - 3
Site A - FF - 3
Site B - NF - 1 (excluded)
Site B - FF - 1
Site B - NF - 2
Site B - FF - 2
Site B - NF - 3
Site B - FF - 3
Site C - NF - 1
Site C - FF - 1
Site C - NF - 2
Site C - FF - 2
Site C - NF - 3
Site C - FF - 3
Site D - NF - 1
Site D - FF - 1
Site D - NF - 2
Site D - FF - 2
Site D - NF - 3
Site D - FF - 3
10
1
0.1
0.01
07:00
08:00
09:00
10:00
11:00
12:00
13:00
14:00
15:00
16:00
10^0
10^1
10^2
10^3
10^4
10^5
10^6
$dN/d\log D_p$ [cm^{-3}]

Figure S5. Contour plots that show particle size distribution over time from all days of sampling (Sites A-D). Each is a compilation of data from scanning mobility and optical particle sizers. NF measurements are on the left and FF measurements are on the right. The x-axis is time of day (HH:MM). The y-axis is particle size (μm). Some plots have blank regions (such as Site A – NF – 1 and Site B – FF – 1) due to instrument malfunction or battery depletion.

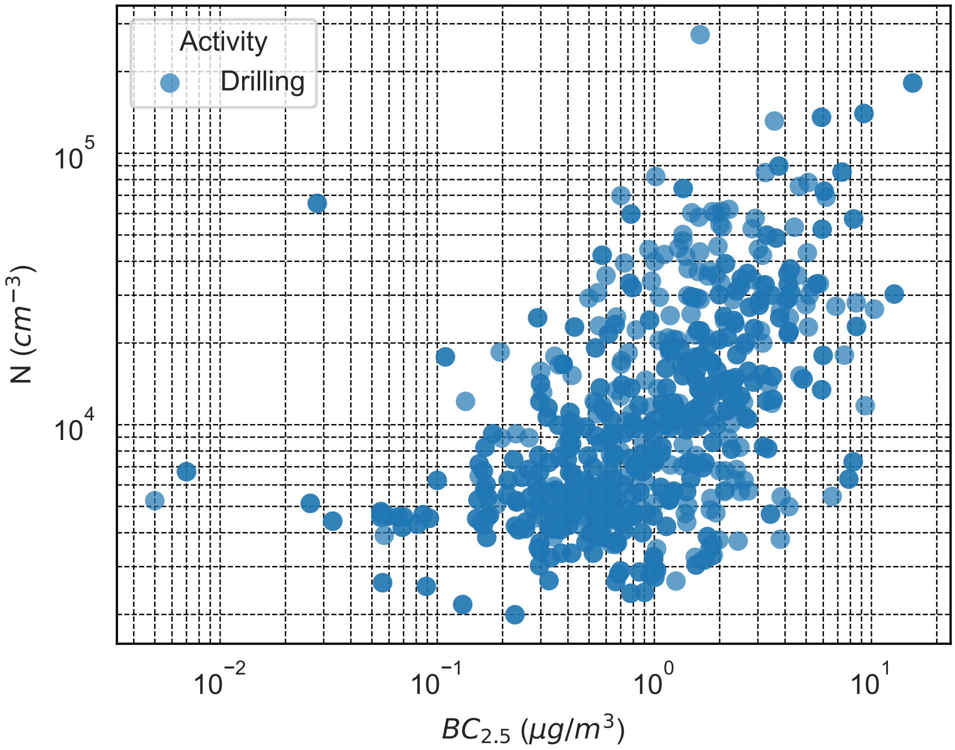

Figure S6. Log-log plot of total particle number concentration versus BC concentration at site C.

Table S1. Average daily (until 16:00) temperatures, wind velocities, and wind directions. Data was retrieved from dmi.dk/vejrarkiv. Gust refers to the maximum wind velocity.

Site	Date	Wind (m/s)	Gust (m/s)	Direction	Temp. (°C)
A	11-Oct-22	5.5	11.0	E	13.3
A	12-Oct-22	2.4	5.5	N	13.4
A	13-Oct-22	2.3	5.4	N	13.2
B	15-Nov-22	6.6	11.8	W	9.8
B	16-Nov-22	8.2	16.4	W	8.3
B	17-Nov-22	8.1	17.7	W	5.7
C	29-Nov-22	5.0	11.1	W	5.7
C	30-Nov-22	2.8	7.1	W	4.3
C	1-Dec-22	2.0	5.6	W	4.4
D	4-Oct-22	3.7	8.0	E/NE	16.1
D	5-Oct-22	4.7	6.4	NE	15.8
D	6-Oct-22	8.2	14.5	E	16.6

Table S2. Summary of online and offline measurements. Abbreviations: elemental carbon (EC), organic carbon (OC), total carbon (TC), black carbon (BC), total particle number measured via SMPS (TPN-SMPS), total particle number measured via DISCMINI (TPN-DM), near-field (NF), far-field (FF), ground worker (GW), non-drill-rig machine operator (MO), drilling rig operator (DO), arithmetic mean (AM), arithmetic standard deviation (ASD), geometric mean (GM), geometric standard deviation (GSD), n (number of days sampled), t (total time in hours), and NA (not applicable).

Site	Position	Activity	EC<br>μg/m³	OC<br>μg/m³	EC/TC	BC<br>μg/m³	TPN-SMPS<br>10³/cm³	TPN-DM<br>10³/cm³	PM2.5<br>μg/m³	PM4<br>μg/m³	PM10<br>μg/m³
A	NF (n=3)	All Day	AM = 1.5<br>ASD = 0.6<br>t = 22.3	AM = 5.6<br>ASD = 1.4<br>t = 22.3	0.21	AM = 1.1<br>ASD = 1.3<br>GM = 0.44<br>GSD = 7.2<br>t = 22.1	AM = 21.7<br>ASD = 37.9<br>GM = 13.6<br>GSD = 2.4<br>t =19.1	AM = 17.8<br>ASD = 31.1<br>GM = 11.3<br>GSD = 2.3<br>t = 16.5	AM = 12.2<br>ASD = 26.9<br>GM = 9.6<br>GSD = 1.6<br>t = 22.2	AM = 15.2<br>ASD = 27.0<br>GM = 12.6<br>GSD = 1.6<br>t = 22.2	AM = 19.1<br>ASD = 27.4<br>GM = 16.4<br>GSD = 1.5<br>t = 22.2
A	FF (n=3)	All Day	AM = 1.1<br>ASD = 0.2<br>t = 22.3	AM = 4.0<br>ASD = 0.8<br>t = 22.3	0.21	AM = 1.2<br>ASD = 1.2<br>GM = 0.75<br>GSD = 3.0<br>t = 22.0	AM = 15.4<br>ASD = 15.2<br>GM = 10.9<br>GSD = 3.5<br>t = 22.1	AM = 13.8<br>ASD = 12.7<br>GM = 10.6<br>GSD = 2.0<br>t = 16.9	AM = 6.9<br>ASD = 4.9<br>GM = 6.2<br>GSD = 1.5<br>t = 18.3	AM = 8.4<br>ASD = 5.0<br>GM = 7.7<br>GSD = 1.4<br>t = 18.3	AM = 19.1<br>ASD = 27.4<br>GM = 16.4<br>GSD = 1.5<br>t = 18.3
A	GW* (n=3)<br><br>*Ground worker operating a drill	All Day	AM = 2.9<br>ASD = 0.4<br>t = 22.1	AM = 57<br>ASD = 5<br>t = 22.1	0.049	NA	NA	AM = 14.5<br>ASD = 53.8<br>GM = 7.7<br>GSD = 2.9<br>t = 18.3	NA	NA	NA
A	DO (n=2)	All Day	AM = 3.3<br>ASD = 0.9<br>t = 16.2	AM = 78<br>ASD = 15<br>t = 16.2	0.040	NA	NA	AM = 38<br>ASD = 118<br>GM = 13.8<br>GSD = 3.3<br>t = 22.0	NA	NA	NA
A	MO-1 (n=3)	All Day	AM = 1.0<br>ASD = 0.4<br>t = 22.4	AM = 15<br>ASD = 2<br>t = 22.4	0.064	NA	NA	AM = 8.9<br>ASD = 17.8<br>GM = 5.9<br>GSD = 2.1<br>t = 11.6	NA	NA	NA
A	MO-2 (n=3)	All Day	AM = 2.1<br>ASD = 0.3<br>t = 21.3	AM = 41<br>ASD = 4<br>t = 21.3	0.049	NA	NA	AM = 13.1<br>ASD = 28.3<br>GM = 7.7<br>GSD = 2.5<br>t = 21.7	NA	NA	NA
A	NF	Drilling	NA	NA	NA	AM = 1.5<br>ASD = 1.4<br>GM = 1.0<br>GSD = 3.1<br>t = 9.6	AM = 32<br>ASD = 50<br>GM = 20.8<br>GSD = 2.2<br>t = 10	AM = 23.4<br>ASD = 36.9<br>GM = 15.4<br>GSD = 2.3<br>t = 7.5	AM = 15.4<br>ASD = 36.5<br>GM = 11.1<br>GSD = 1.8<br>t = 10.0	AM = 18.3<br>ASD = 36.5<br>GM = 14.2<br>GSD = 1.7<br>t = 10.0	AM = 22.2<br>ASD = 36.7<br>GM = 18.0<br>GSD = 1.6<br>t = 10.0
A	FF	Drilling	NA	NA	NA	AM = 1.3<br>ASD = 1.2<br>GM = 0.86<br>GSD = 2.7<br>t =9.5	AM = 20<br>ASD = 18<br>GM = 15.8<br>GSD = 1.9<br>t = 10	AM = 17.1<br>ASD = 15.0<br>GM = 13.7<br>GSD = 1.9<br>t = 7.7	AM = 7.5<br>ASD = 6.2<br>GM = 6.6<br>GSD = 1.6<br>t = 10.0	AM = 8.9<br>ASD = 6.3<br>GM = 8.0<br>GSD = 1.5<br>t = 10.0	AM = 10.7<br>ASD = 6.5<br>GM = 9.8<br>GSD = 1.5<br>t = 10.0
A	NF	Pause	NA	NA	NA	AM = 0.57<br>ASD = 0.59<br>GM = 0.35<br>GSD = 3.7<br>t =2.6	AM = 5.3<br>ASD = 2.2<br>GM = 4.6<br>GSD = 1.9<br>t = 2.8	AM = 4.5<br>ASD = 1.1<br>GM = 4.3<br>GSD = 1.4<br>t = 2.0	AM = 7.9<br>ASD = 2.0<br>GM = 7.6<br>GSD = 1.3<br>t = 2.8	AM = 10.9<br>ASD = 2.8<br>GM = 10.5<br>GSD = 1.3<br>t = 2.8	AM = 15.0<br>ASD = 5.4<br>GM = 14.4<br>GSD = 1.3<br>t = 2.8
A	FF	Pause	NA	NA	NA	AM = 0.48<br>ASD = 0.26<br>GM = 0.37<br>GSD = 2.6<br>t = 2.5	AM = 5.7<br>ASD = 2.5<br>GM = 4.8<br>GSD = 1.9<br>t = 2.8	AM = 4.7<br>ASD = 1.0<br>GM = 4.6<br>GSD = 1.2<br>t = 2.0	AM = 5.5<br>ASD = 1.2<br>GM = 5.3<br>GSD = 1.3<br>t = 2.8	AM = 7.0<br>ASD = 1.5<br>GM = 6.8<br>GSD = 1.3<br>t = 2.8	AM = 8.7<br>ASD = 1.9<br>GM = 8.4<br>GSD = 1.3<br>t = 2.8
B	NF (n=2)	All Day	AM = 3.8<br>ASD = 1.9<br>t = 15.6	AM = 11<br>ASD = 4<br>t = 15.6	0.26	AM = 4.6<br>ASD = 5.7<br>GM = 1.7<br>GSD = 7.3<br>t = 15.6	AM = 67<br>ASD = 124<br>GM = 32<br>GSD = 3.4<br>t = 15.6	AM = 44<br>ASD = 79<br>GM = 22<br>GSD = 3.3<br>t = 4.9	AM = 28<br>ASD = 45<br>GM = 13<br>GSD = 3<br>t = 15.6	AM = 35<br>ASD = 52<br>GM = 20<br>GSD = 3<br>t = 15.6	AM = 68<br>ASD = 111<br>GM = 38<br>GSD = 3<br>t = 15.6
B	FF (n=3)	All Day	AM = 0.7<br>ASD = 0.5<br>t = 24.2	AM = 4.5<br>ASD = 2.8<br>t = 24.2	0.13	AM = 0.75<br>ASD = 0.75<br>GM = 0.4<br>GSD = 3<br>t = 23.2	AM = 5.7<br>ASD = 4.5<br>GM = 4.2<br>GSD = 2.1<br>t = 23.5	AM = 7.2<br>ASD = 6.3<br>GM = 5.5<br>GSD = 2.0<br>t = 10.2	AM = 7.8<br>ASD = 7.9<br>GM = 4.5<br>GSD = 2.8<br>t = 23.5	AM = 9.6<br>ASD = 10.6<br>GM = 6.1<br>GSD = 2.5<br>t = 23.5	AM = 15.8<br>ASD = 35<br>GM = 10.1<br>GSD = 2.3<br>t = 23.5
B	DO (n=3)	All Day	AM = 2.8<br>ASD = 0.9<br>t = 24.3	AM = 63<br>ASD = 22<br>t = 24.3	0.045	NA	NA	AM = 53.3<br>ASD = 117<br>GM = 12.1<br>GSD = 5.3<br>t = 23.4	NA	NA	NA
B	MO-1 (n=3)	All Day	AM = 3.7<br>ASD = 1.0<br>t = 23.6	AM = 32<br>ASD = 13<br>t = 23.6	0.12	NA	NA	AM = 47.1<br>ASD = 185<br>GM = 15.1<br>GSD = 3.0<br>t =19.8	NA	NA	NA
B	MO-2 (n=2)	All Day	AM = 2.6<br>ASD = 1.3<br>t = 15.7	AM = 62<br>ASD = 22<br>t = 15.7	0.039	NA	NA	AM = 158<br>ASD = 604<br>GM = 15.8<br>GSD = 6.1<br>t = 10.7	NA	NA	NA
B	NF	Drilling	NA	NA	NA	AM = 7.4<br>ASD = 6.6<br>GM = 4.2<br>GSD = 4.2<br>t = 6.1	AM = 78<br>ASD = 164<br>GM = 35<br>GSD = 3.5<br>t = 6.1	AM = 17.1<br>ASD = 13.4<br>GM = 12.5<br>GSD = 2.4<br>t = 1.5	AM = 36<br>ASD = 54<br>GM = 17<br>GSD = 3.3<br>t = 6.1	AM = 43<br>ASD = 57<br>GM = 24<br>GSD = 2.9<br>t = 6.1	AM = 75<br>ASD = 92<br>GM = 45<br>GSD = 2.7<br>t = 6.1
B	FF	Drilling	NA	NA	NA	AM = 0.58<br>ASD = 0.70<br>GM = 0.31<br>GSD = 2.86<br>t = 6.3	AM = 4.5<br>ASD = 4.7<br>GM = 3.1<br>GSD = 2.2<br>t = 6.1	AM = 8.3<br>ASD = 7.5<br>GM = 6.4<br>GSD = 2.0<br>t = 3.6	AM = 5.5<br>ASD = 7.0<br>GM = 3.3<br>GSD = 2.5<br>t = 8.1	AM = 7.2<br>ASD = 7.9<br>GM = 4.7<br>GSD = 2.3<br>t = 8.1	AM = 13<br>ASD = 20<br>GM = 8.4<br>GSD = 2.2<br>t = 8.1
B	NF	Welding	NA	NA	NA	AM = 1.55<br>ASD = 2.54<br>GM = 0.66<br>GSD = 7.33<br>t = 2.2	AM = 61<br>ASD = 80<br>GM = 30<br>GSD = 3.5<br>t = 2.2	NA	AM = 21.5<br>ASD = 31.4<br>GM = 10.8<br>GSD = 3.1<br>t =2.23	AM = 36<br>ASD = 63<br>GM = 18<br>GSD = 3.1<br>t = 2.23	AM = 93<br>ASD = 208<br>GM = 37<br>GSD = 3.4<br>t = 2.23
B	FF	Welding	NA	NA	NA	AM = 0.15<br>ASD = 0.039<br>GM = 0.146<br>GSD = 1.26<br>t = 2.1	AM = 3.4<br>ASD = 2.8<br>GM = 2.7<br>GSD = 2.0<br>t = 2.2	NA	AM = 1.9<br>ASD = 0.8<br>GM = 1.8<br>GSD = 1.3<br>t = 2.23	AM = 3.6<br>ASD = 3.7<br>GM = 3.1<br>GSD = 1.6<br>t = 2.23	AM = 11<br>ASD = 20<br>GM = 6.8<br>GSD = 2.0<br>t = 2.23
B	NF	Pause	NA	NA	NA	AM = 0.78<br>ASD = 1.03<br>GM = 0.129<br>GSD = 20.9<br>t = 0.65	AM = 5.9<br>ASD = 5.8<br>GM = 4.1<br>GSD = 2.3<br>t = 0.65	NA	AM = 6.3<br>ASD = 5.6<br>GM = 5.4<br>GSD = 2.2<br>t = 0.65	AM = 14.4<br>ASD = 16.8<br>GM = 9.4<br>GSD = 2.4<br>t = 0.65	AM = 39<br>ASD = 53<br>GM = 23<br>GSD = 2.7<br>t = 0.65
B	FF	Pause	NA	NA	NA	AM = 0.135<br>ASD = 0.035<br>GM = 0.131<br>GSD = 1.33<br>t = 0.58	AM = 3.0<br>ASD = 3.3<br>GM = 2.4<br>GSD = 1.8<br>t = 0.65	NA	AM = 2.2<br>ASD = 2.3<br>GM = 1.8<br>GSD = 1.6<br>t = 0.65	AM = 4.1<br>ASD = 4.5<br>GM = 3.2<br>GSD = 1.8<br>t = 0.65	AM = 13<br>ASD = 23<br>GM = 7.4<br>GSD = 2.2<br>t = 0.65
C	NF (n=3)	All Day	AM = 1.8<br>ASD = 0.2<br>t = 25.6	AM = 5.6<br>ASD = 1.2<br>t = 25.6	0.24	AM = 1.1<br>ASD = 1.5<br>GM = 0.42<br>GSD = 6.9<br>t = 25.2	AM = 18<br>ASD = 50<br>GM = 9.7<br>GSD = 2.4<br>t = 25.1	AM = 26<br>ASD = 120<br>GM = 6.0<br>GSD = 3.7<br>t = 10.1	AM = 15<br>ASD = 13<br>GM = 13<br>GSD = 1.5<br>t = 25.2	AM = 17<br>ASD = 14<br>GM = 15<br>GSD = 1.5<br>t = 25.2	AM = 24<br>ASD = 22<br>GM = 20<br>GSD = 1.7<br>t = 25.2
C	FF (n=3)	All Day	AM = 1.3<br>ASD = 0.1<br>t = 25.4	AM = 4.3<br>ASD = 2.0<br>t = 25.4	0.26	AM = 1.6<br>ASD = 2.2<br>GM = 0.4<br>GSD = 15.3<br>t = 24.8	AM = 16<br>ASD = 38<br>GM = 6.4<br>GSD = 3.1<br>t = 24.9	AM = 17<br>ASD = 43<br>GM = 5.7<br>GSD = 3.5<br>t = 10.0	AM = 13<br>ASD = 15<br>GM = 10.5<br>GSD = 1.8<br>t = 19.3	AM = 15<br>ASD = 16<br>GM = 12<br>GSD = 1.8<br>t = 19.3	AM = 22<br>ASD = 38<br>GM = 16<br>GSD = 1.9<br>t = 19.3
C	GW (n=3)	All Day	AM = 4.6<br>ASD = 1.6<br>t =24.6	AM = 96<br>ASD = 43<br>t = 24.6	0.052	NA	NA	NA	NA	NA	NA

C	DO (n=3)	All Day	AM = 2.3<br>ASD = 0.9<br>t = 25.5	AM = 67<br>ASD = 35<br>t = 25.5	0.036	NA	NA	AM = 43<br>ASD = 101<br>GM = 6.7<br>GSD = 6.1<br>t =12.9	NA	NA	NA
C	MO (n=3)	All Day	AM = 1.0<br>ASD = 0.8<br>t = 25.4	AM = 26<br>ASD = 28<br>t = 25.4	0.066	NA	NA	AM = 26<br>ASD = 120<br>GM = 6.0<br>GSD = 3.7<br>t = 10.1	NA	NA	NA
C	NF	Drilling	NA	NA	NA	AM = 1.5<br>ASD = 1.8<br>GM = 0.67<br>GSD = 6.2<br>t = 6.7	AM = 16<br>ASD = 19<br>GM = 11.1<br>GSD = 2.2<br>t = 6.5	AM = 21.4<br>ASD = 82.2<br>GM = 6.0<br>GSD = 3.4<br>t =2.6	AM = 15<br>ASD = 6.6<br>GM = 14<br>GSD = 1.4<br>t = 6.5	AM = 17<br>ASD = 7.6<br>GM = 15.5<br>GSD = 1.4<br>t = 6.5	AM = 22.4<br>ASD = 14<br>GM = 20<br>GSD = 1.6<br>t = 6.5
C	FF	Drilling	NA	NA	NA	AM = 1.7<br>ASD = 2.0<br>GM = 0.47<br>GSD = 12<br>t = 4.67	AM = 14.6<br>ASD = 24.6<br>GM = 6.9<br>GSD = 3.0<br>t = 6.5	AM = 14.6<br>ASD = 31.7<br>GM = 5.4<br>GSD = 3.3<br>t = 2.6	AM = 11.3<br>ASD = 9.8<br>GM = 9.3<br>GSD = 1.8<br>t = 4.5	AM = 13.5<br>ASD = 12.5<br>GM = 11<br>GSD = 1.8<br>t = 4.5	AM = 22<br>ASD = 39<br>GM = 15<br>GSD = 2.0<br>t = 4.5
C	NF	Welding	NA	NA	NA	AM = 0.53<br>ASD = 0.56<br>GM = 0.28<br>GSD = 5.4<br>t = 1.23	AM = 17.6<br>ASD = 8.7<br>GM = 15.6<br>GSD = 1.7<br>t = 1.2	AM = 9.5<br>ASD = 5.1<br>GM = 7.8<br>GSD = 2.0<br>t = 0.25	AM = 12.5<br>ASD = 3.3<br>GM = 12.1<br>GSD = 1.3<br>t = 1.2	AM = 14<br>ASD = 4.3<br>GM = 13.7<br>GSD = 1.3<br>t = 1.2	AM = 18.3<br>ASD = 8.9<br>GM = 17<br>GSD = 1.5<br>t = 1.2
C	FF	Welding	NA	NA	NA	AM = 0.45<br>ASD = 0.71<br>GM = 0.080<br>GSD = 14<br>t = 0.917	AM = 82<br>ASD = 77<br>GM = 48<br>GSD = 3.4<br>t = 1.1	AM = 7.2<br>ASD = 16.8<br>GM = 4.2<br>GSD = 2.2<br>t = 0.25	AM = 36<br>ASD = 31<br>GM = 27<br>GSD = 2.1<br>t = 1.0	AM = 37<br>ASD = 31<br>GM = 28<br>GSD = 2.0<br>t = 1.0	AM = 41<br>ASD = 31<br>GM = 32<br>GSD = 1.9<br>t = 1.0
C	NF	Pause	NA	NA	NA	AM = 0.29<br>ASD = 0.38<br>GM = 0.11<br>GSD = 9.1<br>t = 3.1	AM = 5.5<br>ASD = 2.4<br>GM = 5.1<br>GSD = 1.4<br>t = 3.0	AM = 2.4<br>ASD = 2.4<br>GM = 1.9<br>GSD = 1.9<br>t = 0.43	AM = 12.4<br>ASD = 6.0<br>GM = 11.5<br>GSD = 1.4<br>t = 3.0	AM = 14<br>ASD = 8.0<br>GM = 13<br>GSD = 1.5<br>t = 3.0	AM = 17<br>ASD = 12<br>GM = 15<br>GSD = 1.6<br>t = 3.0
C	FF	Pause	NA	NA	NA	AM = 0.97<br>ASD = 0.78<br>GM = 0.33<br>GSD = 10.5<br>t = 2.7	AM = 3.8<br>ASD = 2.0<br>GM = 3.5<br>GSD = 1.5<br>t = 3.0	AM = 4.4<br>ASD = 4.6<br>GM = 3.6<br>GSD = 1.7<br>t = 0.67	AM = 8.9<br>ASD = 4.4<br>GM = 8.2<br>GSD = 1.5<br>t = 2.4	AM = 10<br>ASD = 4.6<br>GM = 9.4<br>GSD = 1.5<br>t = 2.4	AM = 14<br>ASD = 9<br>GM = 13<br>GSD = 1.5<br>t = 2.4
D	NF (n=3)	All Day	AM = 1.0<br>ASD = 0.4<br>t = 24.2	AM = 6<br>ASD = 2<br>t = 24.2	0.15	AM = 0.64<br>ASD = 0.56<br>GM = 0.36<br>GSD = 5.2<br>t = 23.3	AM = 5.3<br>ASD = 3.2<br>GM = 4.7<br>GSD = 1.6<br>t = 23.8	AM = 6.8<br>ASD = 29.6<br>GM = 4.6<br>GSD = 1.8<br>t = 16.8	AM = 5.8<br>ASD = 2.8<br>GM = 5.3<br>GSD = 1.5<br>t = 23.8	AM = 8.0<br>ASD = 6.6<br>GM = 7.1<br>GSD = 1.5<br>t = 23.8	AM = 12.9<br>ASD = 24.5<br>GM = 9.9<br>GSD = 1.7<br>t = 23.8
D	FF (n=3)	All Day	AM = 1.0<br>ASD = 0.5<br>t = 24.6	AM = 6.7<br>ASD = 2.9<br>t = 24.6	0.13	AM = 0.56<br>ASD = 0.64<br>GM = 0.17<br>GSD = 11.6<br>t = 23.8	AM = 5.8<br>ASD = 5.7<br>GM = 4.6<br>GSD = 1.9<br>t = 24.1	AM = 6.2<br>ASD = 9.9<br>GM = 5.0<br>GSD = 1.7<br>t = 21.8	AM = 7.0<br>ASD = 4.4<br>GM = 6.5<br>GSD = 1.4<br>t = 24.1	AM = 10.5<br>ASD = 15.4<br>GM = 8.9<br>GSD = 1.5<br>t = 24.1	AM = 20.8<br>ASD = 64.2<br>GM = 12.9<br>GSD = 1.9<br>t = 24.1
D	GW-1 (n=2)	All Day	AM = 3.2<br>ASD = 0.4<br>t = 13.3	AM = 39<br>ASD = 17<br>t = 13.3	0.08	NA	NA	NA	NA	NA	NA
D	GW-2 (n=2)	All Day	AM = 2.8<br>ASD = 0.7<br>t = 10.8	AM = 43<br>ASD = 9<br>t = 10.8	0.060	NA	NA	NA	NA	NA	NA
D	GW-3 (n=1)	All Day	AM = 4.0<br>ASD = NA<br>t = 7.1	AM = 95<br>ASD = NA<br>t = 7.1	0.040	NA	NA	NA	NA	NA	NA
D	MO-1 (n=2)	All Day	AM = 0.5<br>ASD = 0.6<br>t = 12.4	AM = 26<br>ASD = 3<br>t = 12.4	0.017	NA	NA	AM = 8.7<br>ASD = 21.7<br>GM = 4.6<br>GSD = 2.4<br>t = 9.5	NA	NA	NA
D	MO-2 (n=2)	All Day	AM = 0.6<br>ASD = 0.1<br>t = 16.0	AM = 26<br>ASD = 12<br>t = 16.0	0.023	NA	NA	AM = 5.1<br>ASD = 16.5<br>GM = 2.3<br>GSD = 3.2<br>t = 14.2	NA	NA	NA
D	MO-3 (n=1)	All Day	AM = 0.92<br>ASD = NA<br>t = 8.8	AM = 20<br>ASD = NA<br>t = 8.8	0.044	NA	NA	AM = 8.1<br>ASD = 17.4<br>GM = 4.8<br>GSD = 2.1<br>t = 8.3	NA	NA	NA
All	NF (n=11)<br>4 positions	All Day	AM = 1.9<br>ASD = 1.2<br>GM = 1.6<br>GSD = 1.8<br>t = 87.7	AM = 6.5<br>ASD = 2.6<br>GM = 6.1<br>GSD = 1.4<br>t = 87.7	0.23	AM = 2.3<br>ASD = 4.1<br>GM = 0.65<br>GSD = 8.0<br>t = 86.2	AM = 24.3<br>ASD = 66.4<br>GM = 10.6<br>GSD = 3.0<br>t = 83.6	AM = 18.6<br>ASD = 67.0<br>GM = 7.8<br>GSD = 2.9<br>t = 45.9	AM = 14.0<br>ASD = 25.5<br>GM = 9.5<br>GSD = 2.1<br>t = 83.6	AM = 17.4<br>ASD = 28.7<br>GM = 12.2<br>GSD = 2.0<br>t = 83.6	AM = 27.8<br>ASD = 56.3<br>GM = 17.7<br>GSD = 2.2<br>t = 83.6
All	FF (n=12)<br>4 positions	All Day	AM = 1.0<br>ASD = 0.4<br>GM = 0.9<br>GSD = 1.7<br>t = 96.5	AM = 4.9<br>ASD = 2.2<br>GM = 4.4<br>GSD = 1.6<br>t = 96.5	0.17	AM = 0.80<br>ASD = 1.3<br>GM = 0.26<br>GSD = 8.4<br>t = 93.8	AM = 10.8<br>ASD = 21.8<br>GM = 6.0<br>GSD = 2.8<br>t = 93.0	AM = 10.3<br>ASD = 20.6<br>GM = 6.4<br>GSD = 2.3<br>t = 56.0	AM = 8.0<br>ASD = 9.5<br>GM = 6.2<br>GSD = 2.0<br>t = 86.1	AM = 10.2<br>ASD = 13.1<br>GM = 8.0<br>GSD = 1.9<br>t = 86.1	AM = 16.8<br>ASD = 43.5<br>GM = 11.3<br>GSD = 2.0<br>t = 86.1
All	GW (n=11)<br>5 workers	All Day	AM = 3.5<br>ASD = 1.1<br>GM = 3.4<br>GSD = 1.3<br>t = 77.9	AM = 65<br>ASD = 32<br>GM = 59<br>GSD = 1.6<br>t = 77.9	0.05	NA	NA	AM = 14.5<br>ASD = 53.8<br>GM = 7.7<br>GSD = 2.9<br>t = 17.3<br>*1 worker	NA	NA	NA
All	DO (n=8)<br>3 workers	All Day	AM = 2.7<br>ASD = 0.9<br>GM = 2.6<br>GSD = 1.4<br>t = 66.0	AM = 69<br>ASD = 24<br>GM = 64<br>GSD = 1.5<br>t = 66.0	0.04	NA	NA	AM = 44<br>ASD = 117<br>GM = 12.3<br>GSD = 4.2<br>t = 42.3	NA	NA	NA
All	MO (n=19)<br>8 workers	All Day	AM = 1.7<br>ASD = 1.3<br>GM = 1.2<br>GSD = 2.9<br>t = 145.6	AM = 31<br>ASD = 18<br>GM = 25<br>GSD = 2.1<br>t = 145.6	0.05	NA	NA	AM = 35<br>ASD = 238<br>GM = 6.5<br>GSD = 3.6<br>t = 84.0	NA	NA	NA
Drilling sites	NF (n=8)<br>3 positions	All Day	-	-	-	AM = 2.5<br>ASD = 4.3<br>GM = 0.70<br>GSD = 8.3<br>t = 62.9	AM = 31.9<br>ASD = 77.2<br>GM = 14.8<br>GSD = 2.9<br>t = 59.8	AM = 24.7<br>ASD = 79.1<br>GM = 10.2<br>GSD = 3.2<br>t = 30.6	AM = 17.3<br>ASD = 29.5<br>GM = 11.9<br>GSD = 2.0<br>t = 59.8	AM = 21.1<br>ASD = 32.9<br>GM = 15.2<br>GSD = 2.0<br>t = 59.8	AM = 33.8<br>ASD = 63.8<br>GM = 22.2<br>GSD = 2.1<br>t = 59.8
Drilling sites	FF (n=9)<br>3 positions	All Day	-	-	-	AM = 0.9<br>ASD = 1.5<br>GM = 0.3<br>GSD = 6.7<br>t = 70.0	AM = 12.5<br>ASD = 24.9<br>GM = 6.6<br>GSD = 3.1<br>t = 68.8	AM = 12.7<br>ASD = 24.5<br>GM = 7.4<br>GSD = 2.6<br>t = 35.6	AM = 8.4<br>ASD = 10.8<br>GM = 6.0<br>GSD = 2.2<br>t = 61.9	AM = 10.0<br>ASD = 12.1<br>GM = 8.0<br>GSD = 2.0<br>t = 61.9	AM = 15.2<br>ASD = 31.8<br>GM = 10.8<br>GSD = 2.0<br>t = 61.9

Table S3. Diesel-powered machines at each worksite.

Site	Machine	Model	Type of Engine	Load of Engine	Fuel	Average Fuel Use Per Day
A	Drill Rig	Hutte HBR 608-4	97/68 ECV (5)	ISO 9249, 186 kW / 249 HP	B7 diesel	unknown
A	Drill Rig	Volvo ECR 88 Plus; Geotech AB drilling tower	97/68 ECII (2)	ISO 9249, 38,2 kW / 52 HP	B7 diesel	unknown (custom build)
A	Excavator	Volvo EWR 150E	97/68 ECIV (4)	ISO 9249, 105 kW / 141 HP	B7 diesel	unknown
A	Excavator	Kobelco SK85MSR-7	97/68 ECV (5)	ISO 14396, 53,7 kW / 73 HP	B7 diesel	5 - 18 L/h
B	Drill Rig	Liebherr LB20; 56 tons	97/68 ECIII A (3a)	ISO 9249, 270 kW / 362 HP	B7 diesel	16 - 75 L/h
B	Backhoe	Hydrema 906G	97/68 ECV (5)	ISO 9249, 90 kW /	B7 diesel	11 - 20 L/h
B	Excavator	Yanmar Vio80	97/68 ECIII A (3a)	ISO 9249, 40,7 kW / 54,6 HP	B7 diesel	5 - 18 L/h
C	Drill Rig	Liebherr LB36 XL 410; 126 tons	97/68 ECIV (4)	ISO 9249, 390 kW / 523 HP	B7 diesel	25 - 90 L/h
C	Front Loader	Liebherr 556	97/68 ECIII B (3b)	ISO 9249, 140 kW /	B7 diesel	5 - 25 L/h
C	Excavator	Takeuchi 200 series	97/68 ECV (5)	ISO 9249, 18,2 kW / 24,4 HP	B7 diesel	3 - 5 L/h (TB230)
C	Front Loader	JCB 407	97/68 ECIII A (3a)	ISO 9249, 48 kW / 64 HP	B7 diesel	5 - 20 L/h
C	Excavator	Caterpillar M318D	97/68 ECIII A (3a)	ISO 9249, 124 kW / 169 HP	B7 diesel	11 - 20 L/h
D	Excavator	Caterpillar 335F (with DPF)	97/68 ECV (5)	ISO 9249, kW / 277 HP	B7 diesel	14 - 20 L/h
D	Excavator	Doosan DX 300 LC	97/68 ECIV (4)	ISO 9249, kW / 197 HP	B7 diesel	11 - 22 L/h
D	Excavator	Hydrema MX16	97/68 ECIV (4)	ISO 9249, 122 kW / HP	B7 diesel	11 - 20 L/h
